# GPS2 ameliorates cigarette smoking-induced pulmonary vascular remodeling by modulating the ras-Raf-ERK axis

**DOI:** 10.1186/s12931-024-02831-0

**Published:** 2024-05-16

**Authors:** Ting Hu, Chaohui Mu, Yanmiao Li, Wanming Hao, Xinjuan Yu, Yixuan Wang, Wei Han, Qinghai Li

**Affiliations:** 1 Department of Respiratory and Critical Care Medicine, Qingdao Municipal Hospital, Qingdao University, Qingdao, China; 2 Qingdao Key Lab of Common Diseases, Qingdao Municipal Hospital, University of Health and Rehabilitation Sciences, 5 Donghai Middle Road, Qingdao, 266071 China; 3 Department of Respiratory and Critical Care Medicine, Qingdao Municipal Hospital, University of Health and Rehabilitation Sciences, 5 Donghai Middle Road, Qingdao, 266071 China

**Keywords:** Pulmonary hypertension, Cigarette, GPS2, MAPK, DNA methylation

## Abstract

**Background:**

Mitogen-activated protein kinase (MAPK)signaling-mediated smoking-associated pulmonary vascular remodeling (PVR) plays an important role in the pathogenesis of group 3 pulmonary hypertension (PH). And G protein pathway suppressor 2 (GPS2) could suppress G-protein signaling such as Ras and MAPK, but its role in cigarette smoking -induced PVR (CS-PVR) is unclear.

**Methods:**

An in vivo model of smoke-exposed rats was constructed to assess the role of GPS2 in smoking-induced PH and PVR. In vitro, the effects of GPS2 overexpression and silencing on the function of human pulmonary arterial smooth cells (HPASMCs) and the underlying mechanisms were explored.

**Results:**

GPS2 expression was downregulated in rat pulmonary arteries (PAs) and HPASMCs after CS exposure. More importantly, CS-exposed rats with GPS2 overexpression had lower right ventricular systolic pressure (RVSP), right ventricular hypertrophy index (RVHI), and wall thickness (WT%) than those without. And enhanced proliferation and migration of HPASMCs induced by cigarette smoking extract (CSE) can be evidently inhibited by overexpressed GPS2. Besides, GPS2siRNA significantly enhanced the proliferation, and migration of HPASMCs as well as activated Ras and Raf/ERK signaling, while these effects were inhibited by zoledronic acid (ZOL). In addition, GPS2 promoter methylation level in rat PAs and HPASMCs was increased after CS exposure, and 5-aza-2-deoxycytidine (5-aza) inhibited CSE-induced GPS2 hypermethylation and downregulation in vitro.

**Conclusions:**

GPS2 overexpression could improve the CS-PVR, suggesting that GPS2 might serve as a novel therapeutic target for PH-COPD in the future.

**Graphical Abstract:**

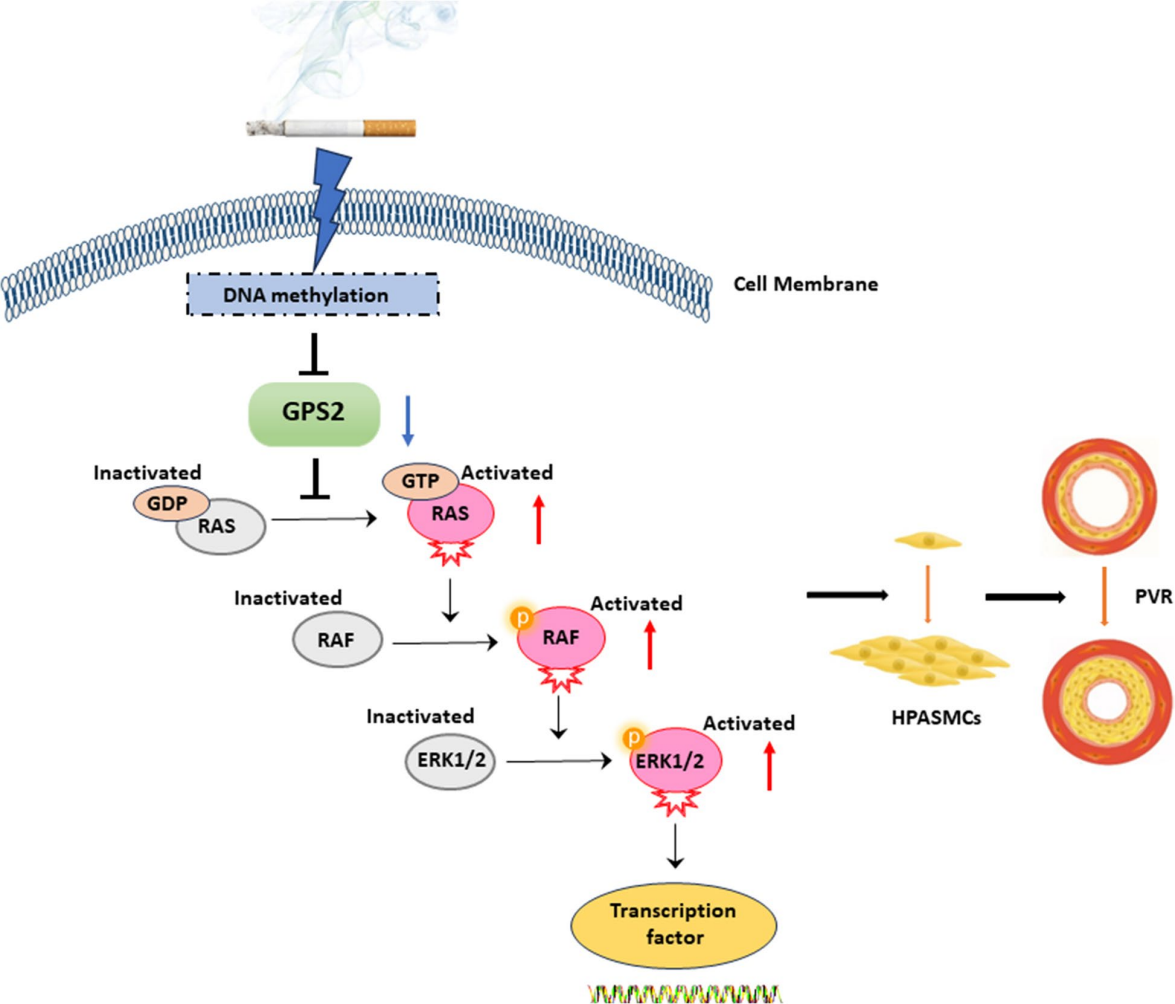

**Supplementary Information:**

The online version contains supplementary material available at 10.1186/s12931-024-02831-0.

## Introduction

Pulmonary hypertension (PH) is defined as a mean pulmonary arterial pressure (mPAP) >20 mmHg at rest [1]. It is accompanied by complex etiologies and high mortality [1]. PH is classified into 5 clinical subgroups, and the group 3 PH (PH due to chronic lung disease (CLD), PH-CLD is a common type [2]. As the most common CLD, chronic obstructive pulmonary disease (COPD) is the 3rd most common cause of death in the world, and its major complication is PH associated with COPD (PH-COPD), which is closely related to acute exacerbation or poor prognosis of COPD patients [2]. Cigarette smoking (CS), a shared risk factor for COPD and PH-COPD [3], could induce airway injury and pulmonary vascular remodeling (PVR) [4, 5]. And advanced COPD patients with severe hypoxemia (about 50–70%) are frequently complicated with mild-moderate PH, while a small part of COPD patients (1–3%) with minor airway damage also have severe PH (mPAP >45mmHg) [1]. Due to the complex pathophysiology of PH-COPD, there is currently no effective drugs but conservative oxygen therapy for PH-COPD.

In general, PVR is the major pathological change of PH-COPD, which is characterized by intimal damage as well as the thickening of the media and adventitia [6, 7]. And the phenotypic changes of pulmonary artery smooth muscle cells (PASMCs) including increased proliferation, enhanced migration and reduced apoptosis, are the pathologic basis of CS-induced medial thickening and PVR [8]. Perivascular inflammation, oxidative stress, senescence, and glycolipid metabolism have been reported to be involved in the occurrence and development of CS-induced PVR (CS-PVR) [9, 10], but the pathogenesis of CS-PVR is still uncertain.

Chronic inflammation caused by CS is a common cause of airway injury and PVR, in which mitogen-activated protein kinase (MAPK) signaling plays a key role [9, 11]. But the specific role of MAPK signaling in CS-PVR has not been fully elucidated. The extracellular signal-regulated kinase (ERK) cascade is one of the classical MAPK signals, consisting of a series of key protein kinases (Ras, Raf, MEK, and ERK). As an important regulatory pathway for modulating smooth muscle function, the ERK pathway has been found to be associated with the occurrence and progression of PH of various types [12–14]. Zhang et al. found that Raf1 expression was significantly elevated in the serum of patients with idiopathic pulmonary arterial hypertension (IPAH) compared with healthy subjects, suggesting that Raf1 might be a potential biomarker of this disease [15]. And inhibition of the Ras/ERK signaling has been shown to improve PVR induced by silymarin in rats [16]. In addition, ERK1/2 pathway activation has been reported to closely linked to CS-PVR and PH development [17, 18]. However, the roles of Ras and Raf1 in CS-PVR have not been fully elucidated and whether they regulate the ERK pathway in CS-PVR is still not clear.

It is well known that ERK1/2 MAPK signaling, which is closely related to CS-PVR, is mainly transduced and regulated by the G protein family [19]. As a repressor of G protein signaling, the G protein pathway suppressor 2 (GPS2) is widely expressed in various mammalian tissues and is involved in many physiological and pathological processes, including metabolism [20–22], immune responses [23], erythroid differentiation [24], and brain development [25]. In addition, GPS2 expression is reduced in a variety of tumors (such as osteosarcoma and melanoma) and adipose tissues, indicating that GPS2 is closely related to tumor cell proliferation and lipid-associated inflammation [26, 27]. Importantly, Zhuang et al. found that GPS2 overexpression upregulated potassium channel protein expression in human embryonic kidney cells by inhibiting ERK1/2^22^. Given that PH is a chronic inflammatory proliferative disease and GPS2 has been reported to play a regulatory role in ERK signaling, we hypothesize that GPS2 may be involved in CS-PVR by regulating the ERK1/2 pathway. However, relevant studies have not been reported yet.

In addition, DNA methylation, as the most common epigenetic modification, is involved in the development of various diseases such as COPD and lung cancer by affecting multigene expression through methylation modification of the genome [28–31]. Currently, most of the studies on DNA methylation in the field of PH have focused on pulmonary arterial hypertension (PAH) [32–36]. However, in 2019, our team was the first to propose CS-PVR, and we revealed that cigarette smoking enhanced RASEF methylation in pulmonary arteries of rats and further induced PVR [37]. And in this study, we will explore whether GPS2 expression is also regulated by DNA methylation after CS exposure.

This study aims to investigate the role of GPS2 in CS-PVR and PH, as well as the possible molecular mechanisms. Our study will further reveal the pathogenesis of CS-PVR, which may provide a new target for the prevention and treatment of PH-COPD.

## Methods

### Animal models

Adult male SD rats were obtained from Jinan Pengyue Laboratory Animal Breeding Company. Rats were randomly divided into a “smoking group” and an “air group”. The “smoking group” was exposed to cigarette smoke from 10 cigarettes (Hong Jin Long, 1.2 mg nicotine, 15 mg tar per cigarette, Wuhan, China) in a plexiglass ventilated box for 1 h each time, two times per day and 5 days per week for 3 months, as previously noted [37]. The “air group” rats were housed in clean air during the same period. After receiving the approval from the Ethics Committee of Qingdao Municipal Hospital, our study was carried out in accordance with the regulations of the Chinese Animal Ethics Committee.

### Preparation of cigarette smoke extract (CSE)

CSE was acquired by combusting Kentucky Research Cigarettes (CODE 3R4F, Class A cigarettes, University of Kentucky, USA). The method for CSE preparation was carried out following previously reported procedures [6, 37] with a few modifications. In brief, CSE was freshly prepared by bubbling the smoke from one research cigarette into 25 ml culture medium, at a burning rate of one cigarette every 5 min. A 0.22 μm filter was used to filter the extract, which was considered to be CSE of 100% concentration.

### Infection with GPS2 overexpressing adeno-associated virus

GPS2 overexpressing adeno-associated virus type 1 (AAV1-GPS2-GFP) and adeno-associated virus type 1-negative control (AAV1-NC-GFP) were both purchased from Hanheng Biotechnology Company. We constructed a smoking-exposed rat model, as described in a previous study [37]. SD rats were divided into the “smoking group” and the “air group”. After 3 months of cigarette exposure, SD rats in the “smoking group” were randomly subdivided into the intervention group and the control group. AAV1-GPS2-GFP (2*10^10^vg/rat) was injected into rats in the intervention group via airway, and thus this group is called “Smoking + AAV1.GPS2” group. And rats in the control group and the “air group” were injected with equivalent doses of AAV1-NC-GFP, which were combined as “AAV1.NC” group. And the control group is termed “Smoking + AAV1.NC” group”. After virus infection, the previous exposure (cigarette smoking or air) was continued for 6 weeks, followed by a hemodynamic assessment. Upon the completion of the treatment, rats were killed and samples were retained. Isolation of rat PAs was performed as previously described [38]. In brief, under the stereo microscope, the isolation of the rat pulmonary arteries was performed. We started to separate the pulmonary arterial trunk which is connected to the right ventricle. We separated the left side into a branch of left pulmonary arteries until the pulmonary hilum. And we separated the right side until the hilum and continuously isolated the arteries downward into the right upper lobe pulmonary artery, the right interlobar pulmonary artery, and the right lower lobe pulmonary artery. The isolated pulmonary arteries were used in subsequent experiments.

### Culture and transfection of HPASMCs

Human pulmonary artery smooth muscle cells (HPASMCs) were purchased from Proximity Life Sciences (China). The cells were cultured in DMEM solution containing 10% fetal bovine serum (FBS) and 1% penicillin/streptomycin and placed in a constant temperature incubator at 37 °C with 5% CO2. HPASMCs were infected with Ad.GPS2 (adenovirus for GPS2 overexpression) and NC.Ad (the control adenovirus) (MOI = 200); and we also transfected HPASMCs with GPS2 siRNA (siGPS2; 50 nM) using Lipofectamine 2000 (Invitrogen, USA) for silencing GPS2. And SiGPS2 target sequences were as follows: the first 5′-CAGCCAGCTTATAGTCCTA − 3′, the second 5′-GGAGAAGCTTTTGGCTCTA − 3′.

### Hemodynamic measurements

Right ventricular systolic pressure (RVSP) was measured by a PowerLab system (AD Instruments, Australia) as a previous study has described [39]. Briefly, animals were anesthetized with intraperitoneal sodium pentobarbital (80 mg/kg), followed by tracheal intubation and invasive ventilation. The right ventricular wall was punctured using a 26G needle (Sigma-Aldrich, St. Louis, MO) connected to a pressure transducer. Data recording was initiated after a stable tracking waveform was obtained, and data were analyzed using Lab Chart software (AD Instruments, Australia).

### Sample processing and assessment of right ventricular hypertrophy

Following the hemodynamic analysis, rat blood samples were collected, and the hearts were dissected. Then, the hearts were flushed with saline to remove residual blood; the atria and valves were excised; and the left and right ventricles were preserved. Finally, the right ventricle (RV) was separated from the left ventricle (LV) and interventricular septum (S). RV hypertrophy was assessed by the weight ratio of RV to (LV + S), which was known as Fulton’s index.

### Assessment of PVR

HE staining was performed for the transverse and longitudinal sections of the rat left lung, and then we assessed the wall thickness of all small pulmonary arteries on the sections that matched the outer diameter (50–150 μm). Compared to pulmonary veins, pulmonary arteries are less dilated, accompanying the airway. They have higher smooth muscle content and thicker walls. These features make them easier to identify. The images were analyzed using Image Pro Plus 6.0 (Media Cybernetics, MD). The wall thickness (WT%) index was calculated to assess PVR (WT% = [(external diameter − internal diameter)/external diameter] × 100%), as a previous study described [40]. 

### Apoptosis assay

After being processed using the methods mentioned above, stimulated HPASMCs were collected. Annexin V-FITC and PI were added to the cell suspensions after incubation for 15 min according to the instructions of the Apoptosis Detection Kit (Elabsience, China). And the level of apoptosis was detected by flow cytometry.

### Migration assay

A transwell migration experiment was conducted using 24-well transwell plates containing chambers with an 8-µm pore size (Transwell, Corning, USA). Migrated HPASMCs were fixed with 4% paraformaldehyde and stained with 0.1% crystal violet, according to the manufacturer’s instructions. Quantification was carried out by counting the number of cells in ten randomly selected fields viewed with a light microscope (Olympus, Tokyo, Japan).

### Immunofluorescence and immunohistochemical staining

Immunofluorescence and immunohistochemical staining were conducted as previously reported [37, 39, 41] with minor changes. And primary antibodies were used as follows : a-SMA (Abcam, Cambridge, UK) and photographed using an orthogonal fluorescence microscope (Olympus, Tokyo, Japan); GPS2 (ProteinTech, Wuhan, China) and photographed using an ordinary light microscope (Olympus, Tokyo, Japan).

### qRT-PCR

Total RNA was extracted from rat pulmonary arteries or HPASMCs by applying Trizol reagent, reverse transcribed, and then subjected to RT-PCR using corresponding kits according to the manufacturer’s instructions (Takara, Japan). And the relative value of mRNA (with GAPDH or β-actin as internal reference) was calculated by the 2^(-ΔΔCt) method.

The primers used were as follows: human β-actin, 5′- AGAAAATCTGGCACCACACCT-3′ (forward) and 5′- GATAGCACAGCCTGGATAGCA-3′ (reverse); rat β-actin, 5′- CGTAAAGACCTCTATGCCAACA-3′ (forward) and 5′- CGGACTCATCGTACTCCTGCT − 3′ (reverse); human GPS2, 5′- CAGCAGAGCCTGACTGTTCA − 3′ (forward) and 5′- AAGCACTTGGGGTCCAAACA − 3′ (reverse); rat GPS2, 5′- AACTGCAGCAGAAGCTTTCA − 3′ (forward) and 5′- GCAGCTGATGTCAGAGTGGT − 3′ (reverse). The ratio for the mRNA of interest was normalized by β-actin.

### Western blot analysis

Total protein was extracted from rat pulmonary arteries or HPASMCs and its concentration was measured by BCA kit (Elabsience, China). Primary antibodies we used were as follows: GPS2 (1:1000), ERK1/2 (1:3500), *p*-ERK1/2 (Thr202/Tyr204) (1:3500) (Wuhan Sanying, China), Raf1 (1:1000), *p*-Raf1 (ser259) (1:1000) (CST, USA), proliferating cell nuclear antigen (PCNA) (1:3000), matrix metalloproteinase 9 (MMP9) (1:1000), TP53 (1:1000) (Abcam, UK), and the relevant secondary antibodies. Finally, ECL luminescent solution was applied to develop the color; a fully automated chemiluminescence gel-imaging analyzing system (Beijing Sage Science and Technology Co., Ltd.) was used for exposure and photography; and Image J software was utilized for analysis. Quantitative results are expressed as gray value ratios of target proteins to GAPDH or β-Actin, except for the signaling pathway proteins, such as “Active-Ras/Ras, p-Raf/Raf, and p-ERK/ERK” which were presented as a ratio of each phosphorylated protein to their respective total proteins.

### Ras activity assay

Ras Pull-Down Activation Assay Biochem Kit (Cat#: 16,117, Thermo Scientific, USA) was applied to rat pulmonary arteries and treated HPASMCs to assess the level of activated Ras. Lastly, ECL luminescent solution was used to develop the color; a fully automated chemiluminescent gel imaging analysis system (Sage Science and Technology, China.) was used for exposure and photography; and Image J software was utilized for analysis.

### DNA methylation sequencing of target regions

DNA was extracted from rat pulmonary arteries or HPASMCs under the combined stimuli (CSE and 5-aza) using the Genomic DNA Extraction Kit (Tiangen Biochemical Technology, China). The extracted DNA was amplified according to the corresponding methylation primers. Subsequently, using Acegen Targeted Methyl Panel Customized System (Acegen Technology, China), DNA sequencing was performed to assess the methylation level of CpGs in its GPS2 promoter. The following methylation primers were used: rat GPS2 5′-TTTYGTTTTTGTTTTTGGTTATTGTGAATTTTAAAG-3′ (forward), 5′-CTATACAAAAAACCCACAACTTCCTC-3′ (reverse); human GPS2 5′-TTGTATGTTTTTTGTTTYGAGGGGATAA-3′ (forward) 5′-CCRTTATAATAAAATTTAAACRTCCTATTCCAC-3′ (reverse).

###  Statistical analysis

GraphPad Prism 8.0 was used for statistical analyses. All quantitative data were expressed as “mean ± standard deviation”. Comparisons between the two groups were performed using Student’s *t* tests. For multiple comparisons, normally distributed data were analyzed using one-way analysis of variance followed by Newman–Keuls tests. A *P*-value < 0.05 was considered statistically significant.

## Results

### A rat model of CS-induced PH

**Fig. 1:**
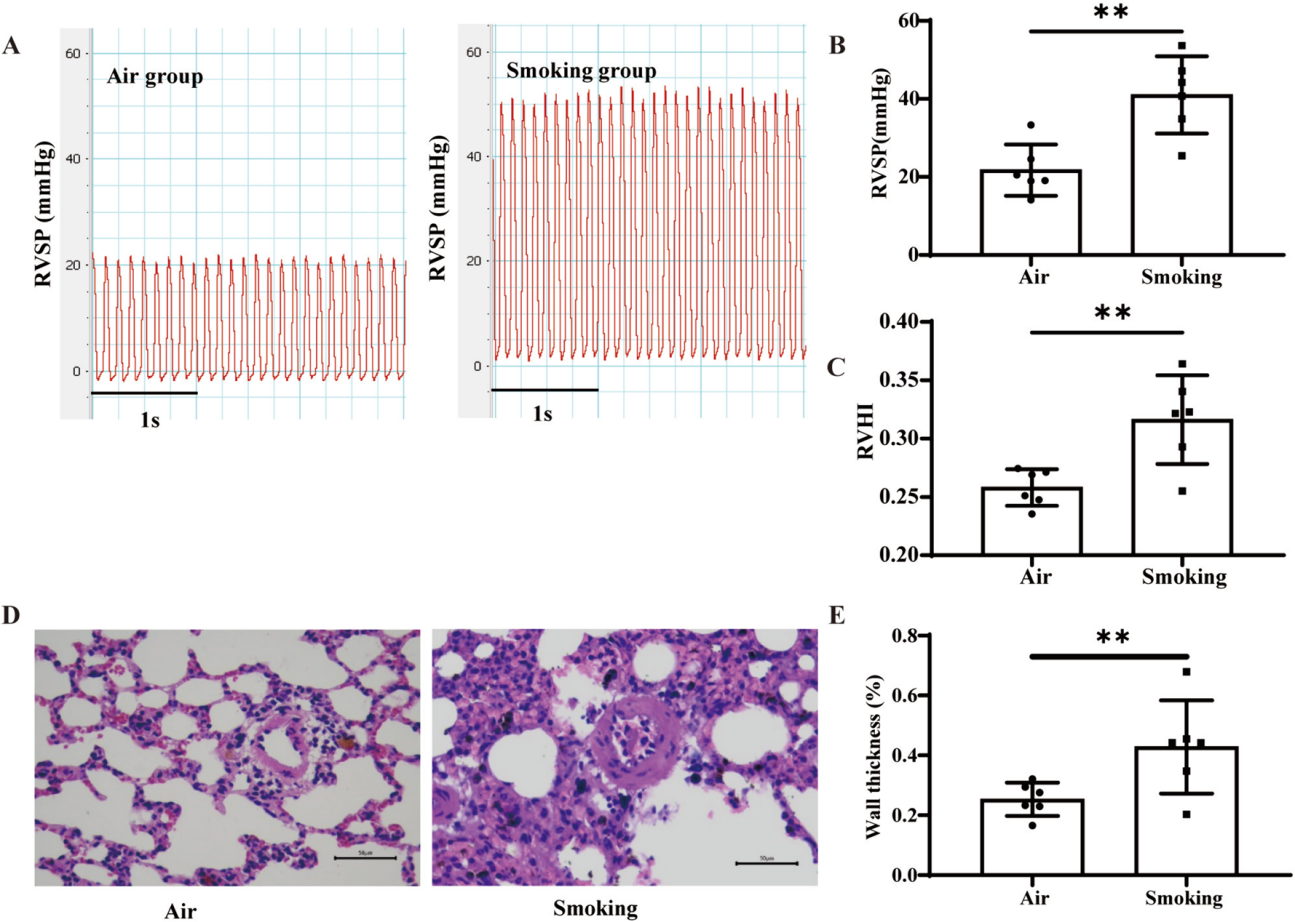
Hemodynamic and histopathological changes in rats.  **A**, **B**  Power Lab system for detection of RVSP in rats; (**C**) right ventricular hypertrophy index in rats; (**D**, **E**) HE staining showing wall thickness of small pulmonary arteries in rats (400X). ( n  = 6, ** *P*  < 0.01, *** *P*  < 0.001; bar = 50 μm)

Compared with the “air group”, the “smoking group” showed significant increases in RVSP (46.84 ± 5.76 mmHg vs. 22.33 ± 6.42 mmHg), right ventricular hypertrophy index (RVHI) and WT%, as well as inflammatory cell infiltration and lung tissue damage (Fig. 1A-E), indicating that the PH model was successfully constructed in the CS-exposed rats.

### CS reduced GPS2 expression in rat PAs and HPASMCs

**Fig. 2:**
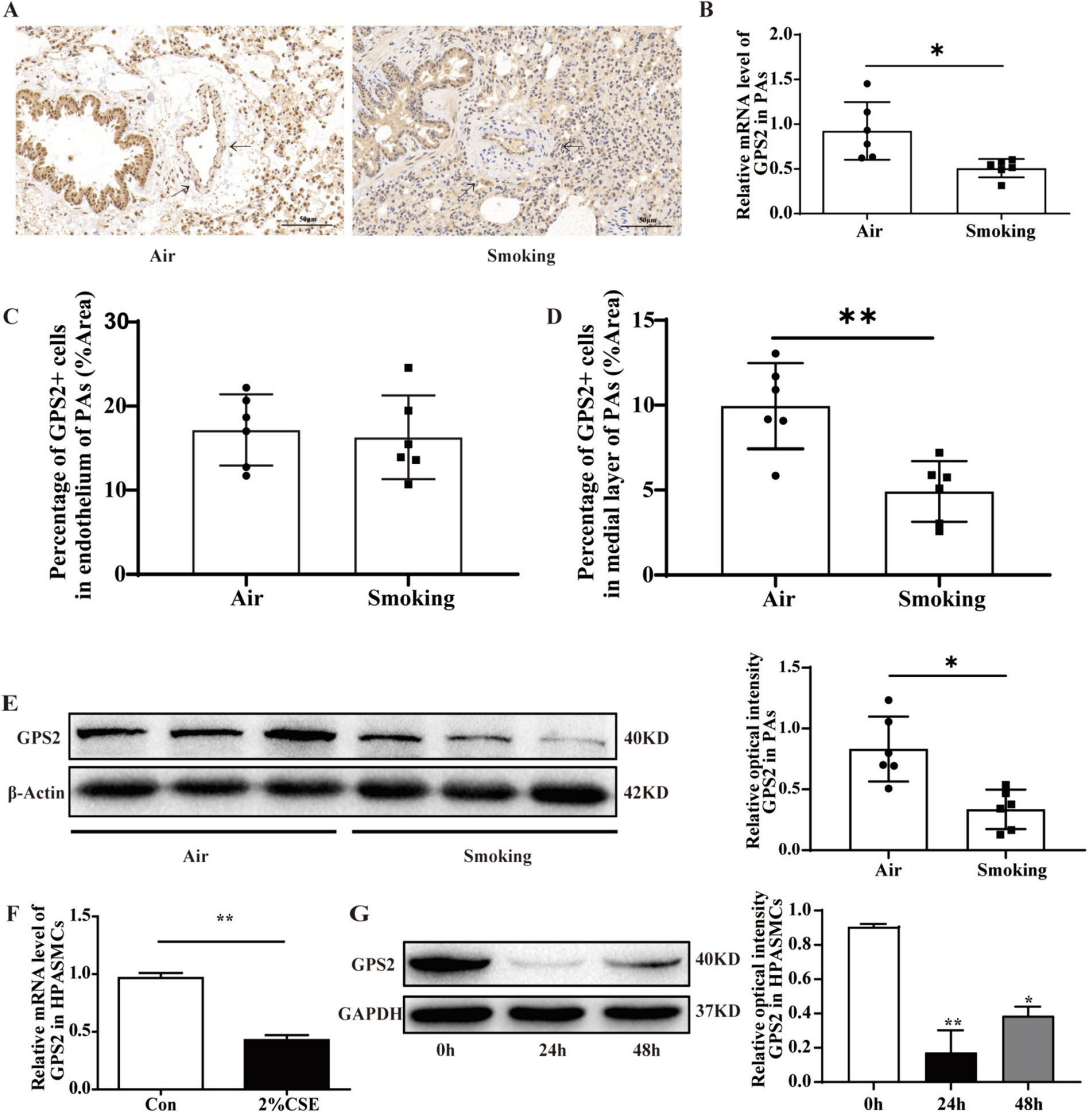
Figure 2 CS exposure affects GPS2 expression in pulmonary artery smooth muscle. **A** Immunohistochemical staining of GPS2 in rat lung tissues (bar=100 μm, arrows indicate positively stained cells in rat PAs); (**B**) qRT-PCR were used to detect GPS2 expression in rat PAs (*n*=6, *P < 0.05); (**C**, **D**) Quantification of GPS2^+^ cells in the intimal and mesial layers of PAs (bar= 100 μm, *n*=6, **P < 0.01). **E** Western blot were used to detect GPS2 expression in rat PAs (*n*=6, *P < 0.05)； (**F**, **G**) qRT-PCR and Western blot were used to detect GPS2 expression in HPASMCs after CSE stimulation; (*n*=3, *P < 0.05, **P < 0.01)

In vivo, the “smoking group” showed significantly decreased GPS2 expression in medial layer of rat PAs compared with the “air group” (Fig. 2A-D). In vitro, 2% CSE significantly promoted the proliferation of HPASMCs (Figure S1). However, GPS2 expression was significantly reduced in the HPASMCs stimulated by 2% CSE for 24–48 h, which was consistent with the changes in vivo (Fig. 2E-G).

### GPS2 overexpression alleviated CS-induced PH in rats

To investigate the effects of GPS2 on CS-PVR and PH in rats, we performed hemodynamic and histopathological assessments of cardiopulmonary tissues by a single transtracheal injection of AAV1.GPS2 in the PH rats after chronic exposure to CS for 3months and continued the exposure for 1.5 months.

**Fig. 3:**
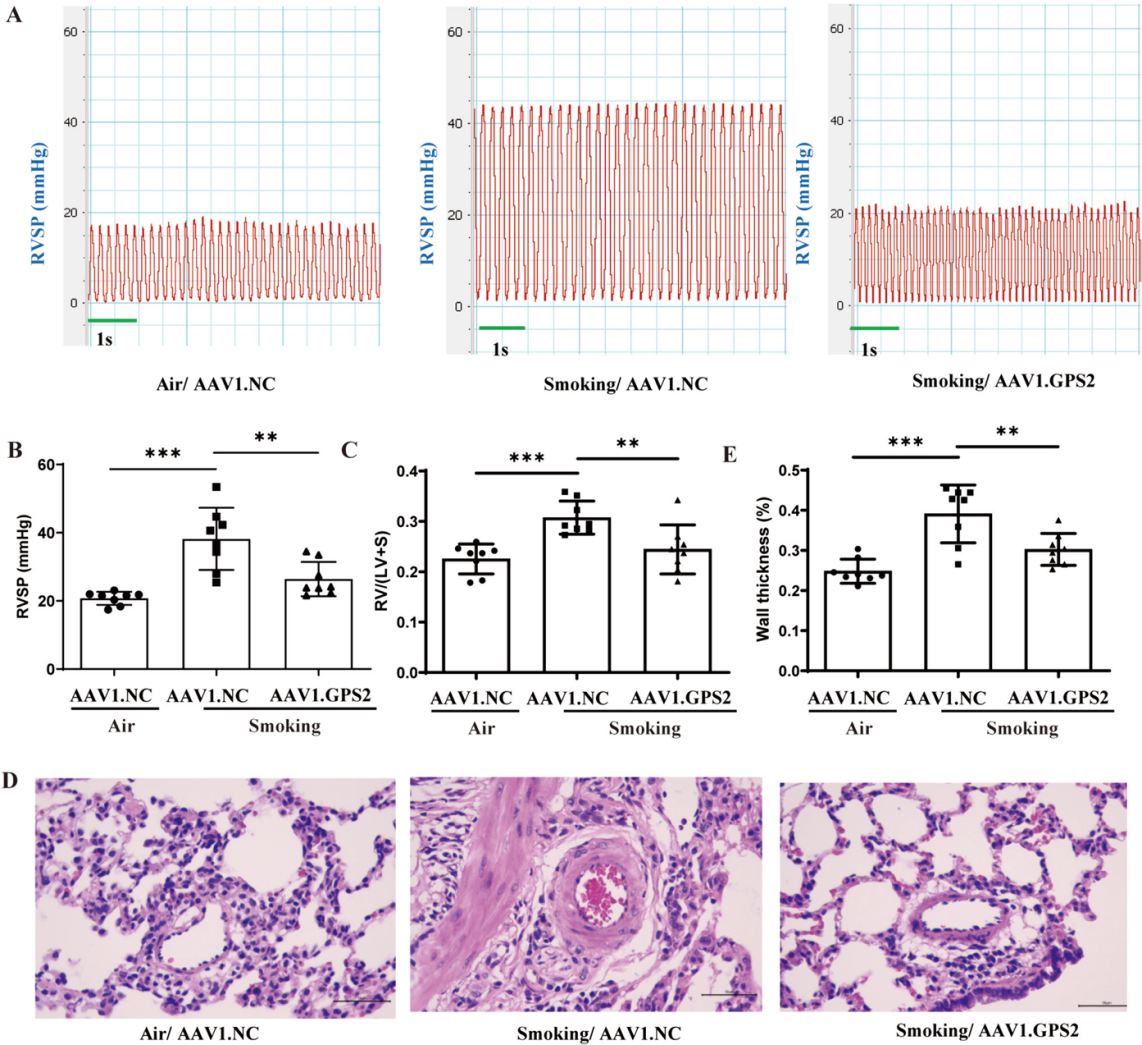
Effects of GPS2 overexpression on hemodynamics and cardiopulmonary structure in CS-exposed rats. **A**, **B** Hemodynamic conditions of rats in the three groups; (**C**) RVHI of rats; (**D**, **E**) HE staining to assess the thickness of pulmonary arterioles vessel wall in rats (400×). (Air+NC.AAV1 group (*n*=8), Smoking+NC.AAV1 group (*n*=8), Smoking+GPS2.AAV1 group (*n*=8), ***P < 0.001; bar= 50 μm)

We found that the “Smoking + AAV1.GPS2 group” had significant increases in the GPS2 expression (Figure S2A-D), as well as decreases in RVSP (23.14 ± 0.99 mmHg vs. 42.03 ± 6.14 mmHg) (Fig. 3A, B), RVHI (Fig. 3C) and WT% (Fig. 3D-E) in the rat PAs, compared with the “Smoking + AAV1.NC” group. Besides, GPS2 overexpression attenuated inflammatory cell infiltration and the lesions of lung tissue in CS-exposed rats (Fig. 3D). This finding indicated that high GPS2 expression alleviated PVR and PH in CS-exposed rats.

### GPS2 overexpression inhibited CSE-induced proliferation and migration of HPASMCs

**Fig. 4:**
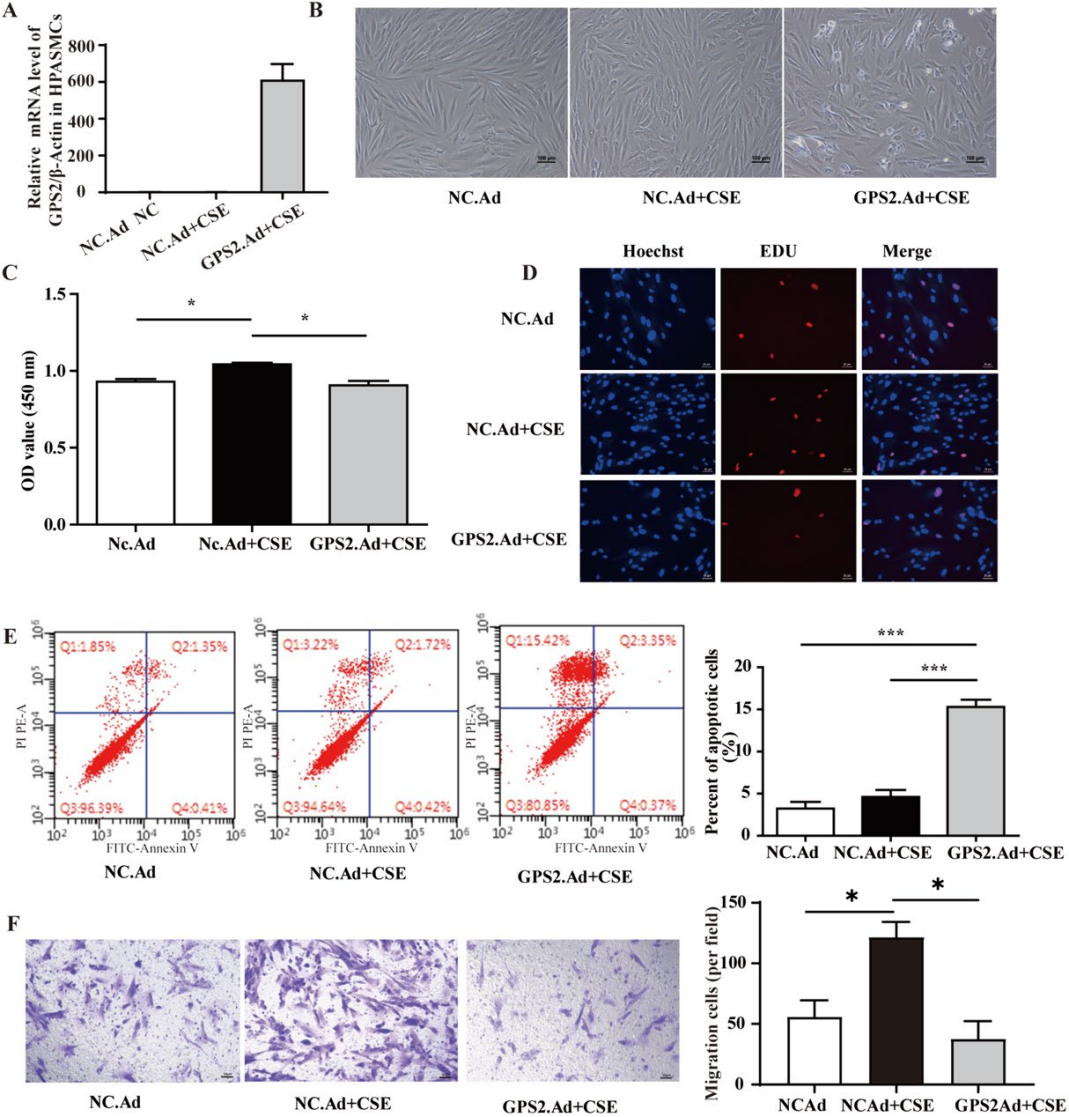
GPS2 overexpression alleviated CSE-induced behavioral changes in HPASMCs. **A** GPS2 expression in GPS2.Ad-infected HPASMCs; (**B-D**) CCK8 and EDU to detect the proliferative ability of HPASMCs; (**E**) Flow assay to detect the apoptosis of HPASMCs; (**F**) Transwell assay to detect the migratory ability of HPASMCs. (*n*=3, *P < 0.05, ***P < 0.001)

To investigate the effect of GPS2 on CSE-induced biological behaviors of HPASMCs, we applied GPS2.Ad in combination with CSE to stimulate HPASMCs and assessed their functional changes after stimulation. qRT-PCR assays showed that GPS2 was successfully overexpressed in HPASMCs (Fig. 4A).

As for proliferation, the cell confluency and cell viability of HPASMCs, as well as the fraction of EDU-positive cells were significantly increased after 48 h of CSE stimulation (Fig. 4B-D), while GPS2.Ad inhibited the CSE-induced HPASMC proliferation. Moreover, Annexin V/PI flow assay revealed that GPS2.Ad increased HPASMC apoptosis (Fig. 4E), while Transwell assay showed that GPS2.Ad also inhibited CSE-induced migration of HPASMCs (Fig. 4F). Next, we evaluated the expression of proteins associated with cell proliferation, apoptosis and migration, and found that CSE increased the expression of PCNA and MMP9 and downregulated TP53 expression in HPASMCs, which could be inhibited by GPS2.Ad (Figure S3).

### GPS2 overexpression inhibited activation of the Ras/Raf/ERK pathway

**Fig. 5:**
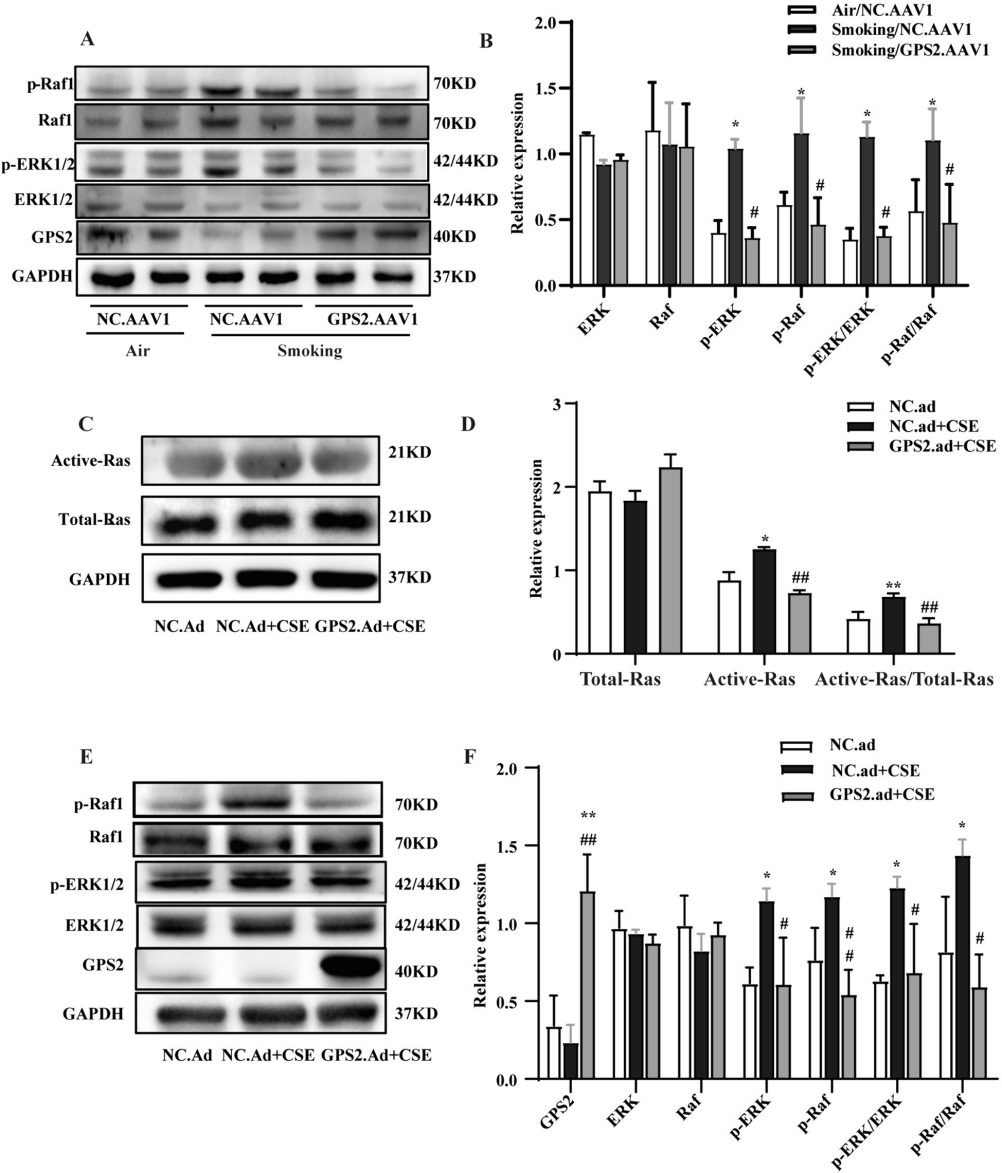
Activation of Ras/Raf1/ERK signaling in PAs of CS-exposed rats and CSE-stimulated HPASMCs. **A**, **B** Western blot to detect Raf1and ERK1/2 activation levels in the PAs of rats. (*n*=8, *P < 0.05 vs Air+NC.AAV1, #*P* < 0.05 vs Smoking+NC.AAV1); (**C**, **D**) Ras pull-down activation assay to determine activated Ras level (*n*=3, *P < 0.05, **P < 0.01); (**E**, **F**) Levels of GPS2 protein and the Ras/MAPK signaling pathway proteins (Ras, Raf1 and ERK1/2) (*n*=3, *P < 0.05, **P < 0.01 vs "NC.Ad "; #*P* < 0.05, ##*P* < 0.01 vs "NC.Ad+CSE")

As mentioned before, we found that GPS2 overexpression in rat pulmonary vessels alleviated CS-induced PVR and PH. To elucidate the possible molecular mechanisms, we performed western blot analysis of potential downstream signaling pathways in rat PAs and HPASMCs. In vivo, rats in the “Smoking + AAV1.GPS2” group showed higher levels of GPS2 protein and significantly lower expression of p-Raf1 and p-ERK1/2 than those in the “Smoking + AAV1.NC” group (Fig. 5A, B). In vitro, we assessed the expression of ERK1/2 MAPK signaling proteins in HPASMCs after being infected with GPS2.Ad and stimulated with 2% CSE. And we found reduced expression of GPS2 protein and activated Ras, Raf1 and ERK1/2 signaling in HPASMCs after being stimulated by CSE, which could be inhibited by GPS2.Ad (Fig. 5C-F). This finding suggested that GPS2 overexpression might improve CS-PVR by regulating Ras/Raf1/ERK signaling in HPASMCs.

### siGPS2-induced proliferation and migration of HPASMCs via Ras/Raf/ERK pathway

To further explore the connection between the inhibitory role of GRPS2 in CS-induced PVR and the Ras/Raf/ERK pathway, we combined zoledronic acid (ZOL) (an inhibitor of Ras signaling) and siGPS2 to stimulate HPASMCs, and then monitored the changes in the biological behaviors of stimulated HPASMCs and the activation of Ras/Raf/ERK pathway in these cells.

**Fig. 6:**
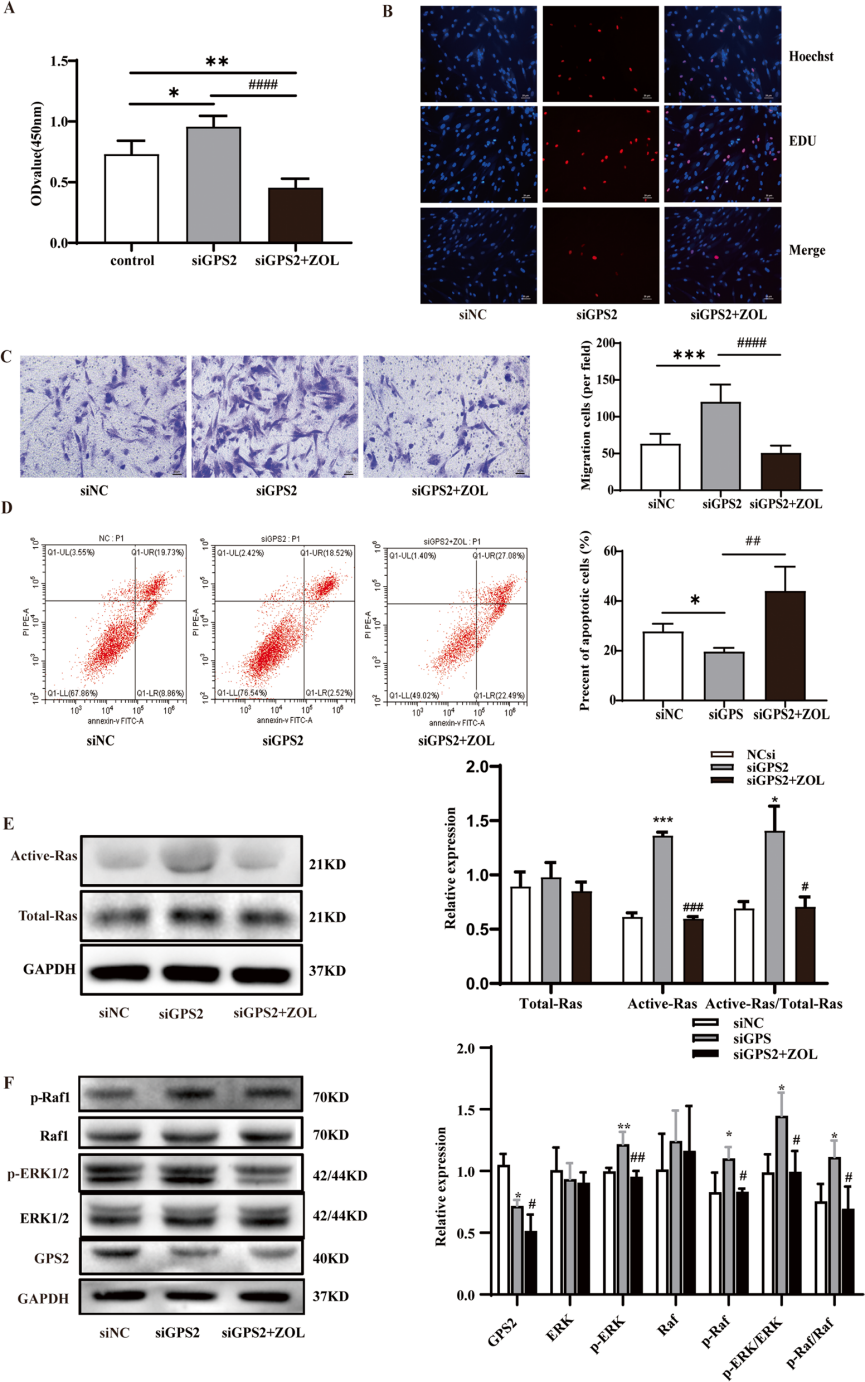
Effects of ZOL on the function of HPASMCs and the activation of Ras/Raf/ERK pathway in the cells. **A**, **B** CCK8 and EDU to detect the proliferative ability of HPASMCs; (**C**) Transwell assay to detect the migration ability of HPASMCs; (**D**) Flow cytometry to detect apoptosis of HPASMCs; (**E**) Ras pull-down activation assay to determine activated Ras level; (**F**) GPS2 protein level and activation levels of Raf1 and ERK1/2. (*n*=3, *P < 0.05, **P < 0.01, ***P < 0.001 vs “siNC”; #*P* < 0.05, ###*P* < 0.001，##*P* < 0.01, ####*P* < 0.0001 vs “siGPS2”)

Our results were as follows. (a) ZOL is not toxic to HPASMCs at a concentration of 100µM or less (Figure S4B), and ZOL at a concentration of 40µM significantly inhibited the activation of Raf1 and ERK1/2 (Figure S4C); (b) siGPS2 promoted cell confluency (Figure S4D), cell viability and the proportion of “EDU-positive cells” in HPASMCs, whereas ZOL inhibited the pro-proliferative effect of siGPS2; (c) siGPS2 increased the migration of HPASMCs, while ZOL inhibited the pro-migratory effect of siGPS2 (Fig. 6A-C); (d) siGPS2 decreased HPASMC apoptosis, whereas ZOL inhibited the anti-apoptotic effect of siGPS2 (Fig. 6D); (e) siGPS2 increased the expression of PCNA and MMP9, as well as decreased TP53 expression, all of which could be reversed by ZOL (Figure S5); (f) siGPS2 attenuated the expression of GPS2 proteins and promoted the activation of Ras, Raf1 and ERK1/2; whereas ZOL inhibited the activation of Ras, Raf1 and ERK1/2 caused by siGPS2 (Fig. 6I-L).

The above results indicated that ZOL (a Ras inhibitor) could inhibit siGPS2-induced proliferation, migration, resistance to apoptosis of HPASMCs, as well as the activation of Ras, Raf1 and ERK1/2 proteins by siGPS2 in the cells.

### CS enhanced *GPS2* promoter methylation in rat PAs and HPASMCs

**Fig. 7:**
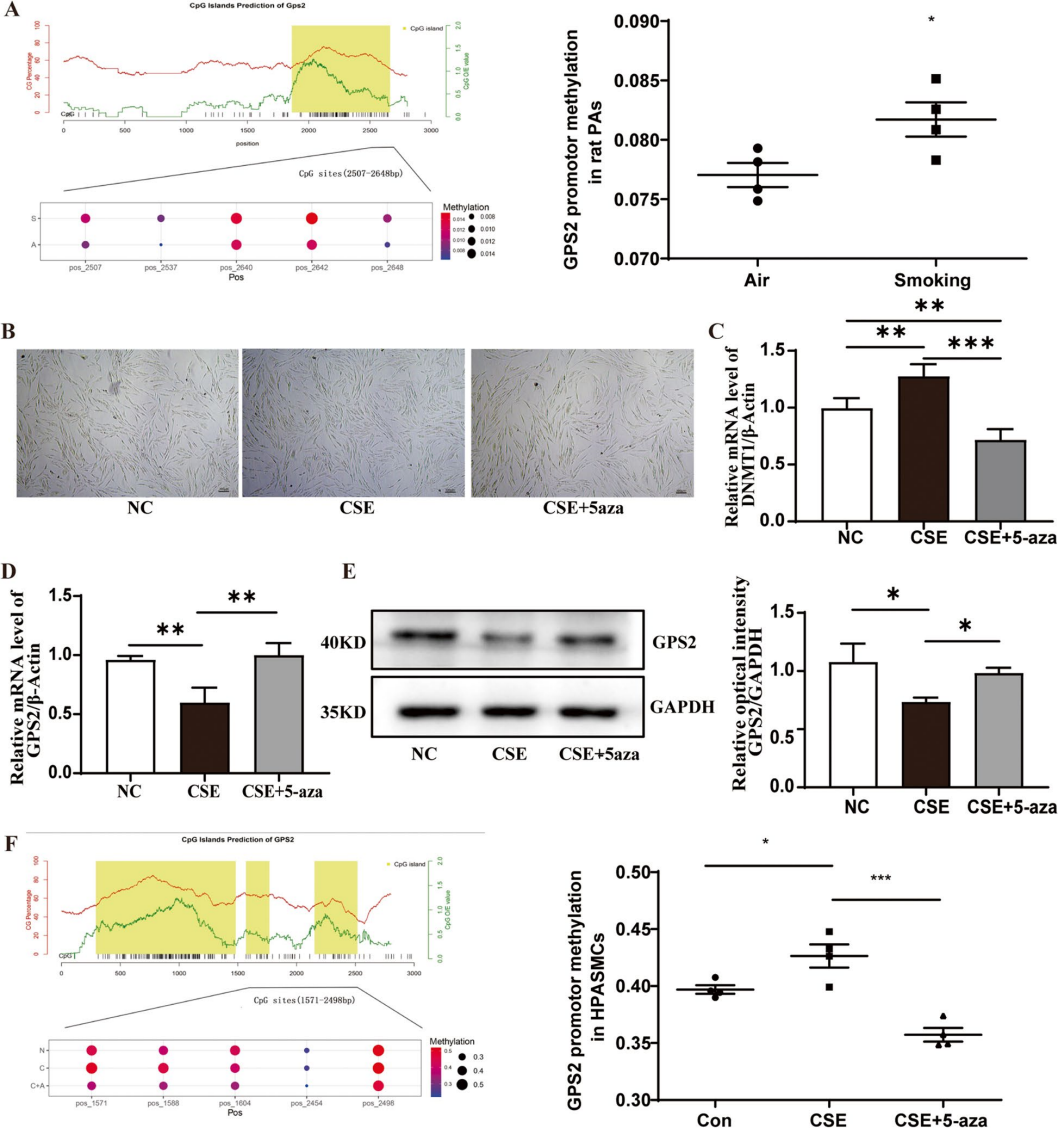
Effect of CS on GPS2 promoter methylation. **A **Targeted bisulfite sequencing (TBS) was used to evaluate the methylation level of GPS2 promoter in rat PAs (*n*=4); (**B**, **C**)the cell confluency of HPASMCs and DNMT1 mRNA expression; (**D**, **E**) qRT-PCR and Western blot were used to assess the expression of GPS2 mRNA and protein in HPASMCs after the combined stimulation by CSE and 5-aza; (**F**) TBS measured the methylation level of GPS2 promoter in HPASMCs after the combined stimulation by CSE and 5-aza. (*n*=3, *P <0.05, **P <0.01, ***P <0.001)

To explore whether DNA methylation is involved in the regulation of GPS2 expression by CS, we conducted methylation sequencing and found that *GPS2* promoter methylation was significantly increased in the PAs of CS-induced PH rats (Fig. 7A). To better understand the effects of DNA methylation on GPS2 expression, we conducted DNA methylation sequence, PCR and WB in vitro. As the result, CSE decreased the cell confluency and increased DNA methylation. But after 5-aza-2-deoxycytidine (5-Aza-CdR), a DNA methyltransferase inhibitor, was added in culture medium of HPASMCs, DNMT1 expression and GPS2 promoter methylation were decreased and GPS2 expression was increased (F7 B-F).

**(A)** Targeted bisulfite sequencing (TBS) was used to evaluate the methylation level of GPS2 promoter in rat PAs (*n* = 4); **(B**, **C)** the cell confluency of HPASMCs and DNMT1 mRNA expression; **(D**, **E)** qRT-PCR and Western blot were used to assess the expression of GPS2 mRNA and protein in HPASMCs after the combined stimulation by CSE and 5-aza; **(F)** TBS measured the methylation level of GPS2 promoter in HPASMCs after the combined stimulation by CSE and 5-aza. (*n* = 3, *P < 0.05, **P < 0.01, ***P < 0.001)

## Discussion

As a common complication of COPD, COPD-associated pulmonary hypertension (PH-COPD) seriously undermines the quality of life and prognosis of COPD patients. Unfortunately, there are currently no effective therapeutic drugs for PH-COPD. Our study is the first to explore the role of GPS2 in CS-PVR and its mechanism using in vivo and in vitro experiments, which might provide a scientific basis for the development of new drugs for PH-COPD. This study yielded several important findings. (1) CS exposure downregulated GPS2 expression in rat PAs and HPASMCs; (2) GPS2 overexpression alleviated smoking-induced PVR and PH in rats, as well as inhibited the proliferation and migration of HPASMCs; (3) ZOL reversed siGPS2-induced proliferation and migration of HPASMCs, as well as the activation of Ras/Raf1/ERK1/2 signaling; and (4) CS exposure increased GPS2 promoter methylation in rat PAs and HPASMCs.

Smoking is a major risk factor for PH-COPD. CS-PVR is an important pathological alteration in PH-COPD, in which the phenotypic switch of pulmonary arterial smooth muscle cells (PASMCs) (excessive proliferation and migration) is the pathological basis of CS-PVR [37, 42, 43]. Experimental evidence from animals has shown that CS-PVR often precedes the occurrence of emphysema [44, 45]. Besides, the historical “vascular hypothesis” for the cause of emphysema has been proposed [46]. Consistently, our study found that RVSP, WT% and RVHI were significantly increased in rats exposed to CS for 3 months without significant alveolar structure changes, indicating that CS-PVR precedes the occurrence of emphysema which usually requires more than 6-month CS-exposure. In addition, Marija Gredic et al. found that myeloid-cell-specific deletion of inducible nitric oxide synthase (iNOS) prevented smoke-induced PH in mice but did not affect hypoxia-induced PH [47]. All the above evidence suggests that the development of CS-PVR is not dependent on emphysema-associated hypoxemia, which may explain the pathophysiological features of cases with mild COPD and severe PH.

G protein-coupled receptors (GPCRs) can participate in the contraction and remodeling of the pulmonary arteries by responding to various extracellular stimuli (including hormones, neurotransmitters, calcium ions, sugars/lipids/proteins/amino acids), and regulating virous signaling pathways such as Ca2+, endothelin, and prostacyclin [48]. As a classical G protein signaling inhibitor, GPS2 has been found to be involved in lipid inflammation and tumorigenesis [49–51]. And PH, a chronic inflammatory proliferative disease of the pulmonary vasculature, might be associated with GPS2. But the role of GPS2 in PH is less investigated. Therefore, our study investigated the role of GPS2 in a CS-induced PH rat model and CSE-stimulated HPASMCs. We found that CS exposure reduced GPS2 expression in rat PAs and HPASMCs and that GPS2 overexpression mitigated the proliferative phenotype of HPASMCs as well as CS-PVR and CS-induced PH in rats, which suggested that CS exposure might induce PVR by downregulating GPS2. These effects of GPS2 on CS-PVR/CS-induced PH might be related to the antiproliferative properties of GPS2 which has been extensively studied in the field of tumors. It has been reported that GPS2 deletion activates AKT signaling to promote proliferation of triple-negative breast cancer cells (MDA-MB-231) [51], and it inhibits the proliferation of gastric cancer cells by increasing the ubiquitylation of the epidermal growth factor receptor (EGFR) [52]. Now the involvement of GPS2 in CS-PVR/CS-induced PH is well established, but the underlying mechanisms remain unclear.

GPS2 was first found in yeast cells and it could inhibit Ras activation [53]. The Ras/Raf/ERK (MAPK) signaling is a key pathway which regulates the inflammation and proliferation in a variety of inflammatory proliferative disorders [54, 55]. Our preliminary study and previous studies from others have found that Raf/ERK signaling is closely associated with the development of IPAH and CS-induced PH [56, 57]. However, whether GPS2 participates in CS-PVR by regulating the Ras/Raf/ERK axis has not been investigated. To explore this, we examined ERK cascade signaling and assessed the effects of the Ras inhibitor-ZOL on the function and related signaling of HPASMCs. We found that CS exposure promoted Raf/ERK1/2 activation in rat PAs and HPASMCs, and that ZOL inhibited siGPS2-induced Ras/Raf/ERK activation as well as the proliferation and migration of HPASMCs. Notably, ZOL is widely used in the clinical treatment of osteoporosis and osteolytic bone disease by inhibiting osteoclast function and survival. Consistent with our study, Hassan’s team reported that ZOL inhibited the proliferation and migration of human aortic smooth muscle cells (HASMCs) [58]. Our finding that ERK cascade signaling played an important role in PVR was also supported by many previous studies [47, 48].

Numerous studies have confirmed that ERK1/2 MAPK activation signaling is closely related to the remodeling phenotype (enhanced proliferation and migration and apoptosis resistance) and the expression of related markers (e.g., PCNA, MMP9, p53, etc.) in pulmonary vascular smooth muscle cells. And the present study demonstrated that GPS2 modulated Ras/Raf/ERK signaling to indirectly affect the expression of p53 and MMP9. In addition, it has been reported that GPS2 can constitute a transcriptional repressor complex directly involved in the transcription of p53^59, 60^, but there is no relevant study on the direct regulation of the transcription of genes such as MMP9 and PCNA.

DNA methylation is the most common epigenetic modification. Environmental exposures, such as chronic cigarette smoking, often alter DNA methylation and participate in gene expression regulation and disease development [61, 62]. To investigate the mechanisms of GPS2 expression in CS-exposed rat PAs and HPASMCs, we evaluated the methylation level of the GPS2 promoter. And we found that CS increased the methylation level of the GPS2 promoter in rat PAs and HPASMCs, while 5-aza (a DNA methylation inhibitor) inhibited CS-induced GPS2 hypermethylation in HPASMCs, which suggested the involvement of “DNA methylation” in the regulation of GPS2 expression by CS. Similarly, our team previously reported that CS enhanced RASEF methylation in rat PAs, contributing to CS-PVR [37]. In addition, aberrant methylation of superoxide dismutase 2 (SOD2) and bone morphogenetic protein receptor 2 (BMPR2) has been found to be involved in the development of PAH [32, 63]. However, unlike the specific treatments taken for other epigenetic abnormalities such as miRNAs, there currently is a lack of DNA methylation therapeutic strategies for PH due to a paucity of gene-specific methylation editing technologies [64, 65].

This study explored the role of GPS2 in CS-PVR and its possible mechanisms using in vivo and in vitro experiments, but it also had some limitations. First, this study focused on the antiproliferative properties of GPS2 in CS-PVR. In addition to pulmonary vascular cell dysfunction, lung inflammation and hypoxia associated with interstitial lung lesions can also trigger PH [66, 67]. In our study, we observed that CS-exposed rats with overexpressed GPS2 exhibited attenuated interstitial lung lesions, suggesting that GPS2 might play a role in pulmonary hypertension by modulating interstitial lung lesions. However, our study did not conduct an animal experiment to verify this finding. Second, The lungs of smoke-exposed rats exhibited extensive infiltration of inflammatory cells, e.g. macrophages and neutrophils [68]. And macrophage activation is closely related to PH [69]. Besides, previous studies have shown that GPS2 expression is associated with macrophage activation [70, 71]. Therefore, it is reasonable to hypothesize that apart from its direct influence on small arteries, GPS2 might also alleviates CS-PH via its interaction with lung macrophages or other immune cells. However, this hypothesis requires further verification. Third, the present study showed that ZOL inhibited Ras/Raf/ERK signaling, as well as suppressed the proliferation and migration of HPASMCs, but its ability to improve CS-PVR and PH needs to be further verified by in vivo animal experiments. Fouth, many studies have found that CS exposure can trigger pulmonary vascular endothelial dysfunction, which is involved in the development of pulmonary hypertension [72, 73]. And the immunohistochemistry in our study suggests that GPS2 is expressed not only in the smooth muscle of pulmonary arteries, but also in the endothelium of the pulmonary vasculature, which suggests that GPS2 may play a role in maintaining endothelial cellular homeostasis. After quantifying GPS2 expression in the pulmonary vascular endothelium, we found no significant difference between the “air group” and the “CS-exposed group”. However, we did not further verify this finding in PAECs.

In summary, our study showed that CS enhanced GPS2 methylation and downregulated its expression, while high GPS2 expression inhibited Ras/Raf1/ERK1/2 activation as well as attenuated CS-PVR and PH. The present study provides new insights into the pathogenesis of PH-COPD and offers a novel target for the drug development in PH-COPD.

### Supplementary Information

Supplementary Material 1.

Supplementary Material 2.

## Data Availability

No datasets were generated or analysed during the current study.

## References

[1] Humbert M, Kovacs G, Hoeper MM, Badagliacca R, Berger RMF, Brida M (2022). 2022 esc/ers guidelines for the diagnosis and treatment of pulmonary hypertension. Eur Heart J.

[2] Hoeper MM, Humbert M, Souza R, Idrees M, Kawut SM, Sliwa-Hahnle K (2016). A global view of pulmonary hypertension. Lancet Respir Med.

[3] Frost AE, Zhao C, Farber HW, Benza R, Yen J, Selej M (2023). Smoking history and pulmonary arterial hypertension: demographics, onset, and outcomes. J Heart Lung Transpl.

[4] Jo HH, Park MJ, Shin HS, Choi HY, Na JB, Choi DS (2019). Adverse effect of smoking on cross-sectional area of small pulmonary vessel and arterial stiffness in healthy smokers without copd. Clin Respir J.

[5] Sun D, Ding D, Li Q, Xie M, Xu Y, Liu X (2021). The preventive and therapeutic effects of aav1-klf4-shrna in cigarette smoke-induced pulmonary hypertension. J Cell Mol Med.

[6] Wu J, Huang Q, Li Q, Gu Y, Zhan Y, Wang T (2022). Increased methyl-cpg-binding domain protein 2 promotes cigarette smoke-induced pulmonary hypertension. Front Oncol.

[7] Santos S, Peinado VI, Ramírez J, Melgosa T, Roca J, Rodriguez-Roisin R (2002). Characterization of pulmonary vascular remodelling in smokers and patients with mild copd. Eur Respir J.

[8] Stenmark KR, Frid MG, Graham BB, Tuder RM (2018). Dynamic and diverse changes in the functional properties of vascular smooth muscle cells in pulmonary hypertension. Cardiovasc Res.

[9] Zhang Y, Xu CB (2020). The roles of endothelin and its receptors in cigarette smoke-associated pulmonary hypertension with chronic lung disease. Pathol Res Pract.

[10] Roger I, Milara J, Belhadj N, Cortijo J. Senescence alterations in pulmonary hypertension. Cells. 2021;10:3456. 10.3390/cells10123456 PMC8700581 34943963

[11] Guan R, Wang J, Li D, Li Z, Liu H, Ding M (2020). Hydrogen sulfide inhibits cigarette smoke-induced inflammation and injury in alveolar epithelial cells by suppressing phd2/hif-1alpha/mapk signaling pathway. Int Immunopharmacol.

[12] Yu MH, Lin MC, Huang CN, Chan KC, Wang CJ (2018). Acarbose inhibits the proliferation and migration of vascular smooth muscle cells via targeting ras signaling. Vascul Pharmacol.

[13] Zhong Y, Feng J, Li J, Fan Z (2017). Curcumin prevents lipopolysaccharide-induced matrix metalloproteinase–2 activity via the ras/mek1/2 signaling pathway in rat vascular smooth muscle cells. Mol Med Rep.

[14] Sun Y, Tian Y, Prabha M, Liu D, Chen S, Zhang R (2010). Effects of sulfur dioxide on hypoxic pulmonary vascular structural remodeling. Lab Invest.

[15] Zhang J, Zhang Y, Li N, Liu Z, Xiong C, Ni X (2009). Potential diagnostic biomarkers in serum of idiopathic pulmonary arterial hypertension. Respir Med.

[16] Zhou C, Chen Y, Kang W, Lv H, Fang Z, Yan F (2019). Mir-455-3p-1 represses fgf7 expression to inhibit pulmonary arterial hypertension through inhibiting the ras/erk signaling pathway. J Mol Cell Cardiol.

[17] Yu M, Liu X, Wu H, Ni W, Chen S, Xu Y (2017). Small interfering rna against erk1/2 attenuates cigarette smoke-induced pulmonary vascular remodeling. Exp Ther Med.

[18] Azevedo PS, Polegato BF, Paiva S, Costa N, Santos P, Bazan S (2021). The role of glucose metabolism and insulin resistance in cardiac remodelling induced by cigarette smoke exposure. J Cell Mol Med.

[19] Goldsmith ZG, Dhanasekaran DN (2007). G protein regulation of mapk networks. Oncogene.

[20] Huang Z, Liang N, Damdimopoulos A, Fan R, Treuter E (2019). G protein pathway suppressor 2 (gps2) links inflammation and cholesterol efflux by controlling lipopolysaccharide-induced atp-binding cassette transporter a1 expression in macrophages. Faseb j.

[21] Sanyal S, Båvner A, Haroniti A, Nilsson LM, Lundåsen T, Rehnmark S (2007). Involvement of corepressor complex subunit gps2 in transcriptional pathways governing human bile acid biosynthesis. Proc Natl Acad Sci U S A.

[22] Zhuang Z, Xiao J, Chen X, Hu X, Li R, Chen S (2018). G protein pathway suppressor 2 enhanced the renal large-conductance ca(2+)-activated potassium channel expression via inhibiting erk1/2 signaling pathway. Am J Physiol Ren Physiol.

[23] Cardamone MD, Krones A, Tanasa B, Taylor H, Ricci L, Ohgi KA (2012). A protective strategy against hyperinflammatory responses requiring the nontranscriptional actions of gps2. Mol Cell.

[24] Ma WB, Wang XH, Li CY, Tian HH, Zhang J, Bi JJ (2020). Gps2 promotes erythroid differentiation by control of the stability of eklf protein. Blood.

[25] Zhang D, Harry GJ, Blackshear PJ, Zeldin DC (2008). G-protein pathway suppressor 2 (gps2) interacts with the regulatory factor x4 variant 3 (rfx4_v3) and functions as a transcriptional co-activator. J Biol Chem.

[26] Shi S, Chen H, Wang H, Wan J, Shi Y, Li J (2023). Genome-wide crispr knockout screening identified g protein pathway suppressor 2 as a novel tumor suppressor for uveal melanoma metastasis. J Cancer Res Clin Oncol.

[27] Toubal A, Clément K, Fan R, Ancel P, Pelloux V, Rouault C (2013). Smrt-gps2 corepressor pathway dysregulation coincides with obesity-linked adipocyte inflammation. J Clin Invest.

[28] Lee KW, Pausova Z (2013). Cigarette smoking and DNA methylation. Front Genet.

[29] Zeng H, Li T, He X, Cai S, Luo H, Chen P (2020). Oxidative stress mediates the apoptosis and epigenetic modification of the bcl-2 promoter via dnmt1 in a cigarette smoke-induced emphysema model. Respir Res.

[30] Tennis MA, Vanscoyk MM, Wilson LA, Kelley N, Winn RA (2012). Methylation of wnt7a is modulated by dnmt1 and cigarette smoke condensate in non-small cell lung cancer. PLoS ONE.

[31] Xu XH, Bao Y, Wang X, Yan F, Guo S, Ma Y, et al. Hypoxic-stabilized epas1 proteins transactivate dnmt1 and cause promoter hypermethylation and transcription inhibition of epas1 in non-small cell lung cancer. Faseb j. 2018;32:694–705. 10.1096/fj.201700715 29920222

[32] Bisserier M, Mathiyalagan P, Zhang S, Elmastour F, Dorfmüller P, Humbert M (2021). Regulation of the methylation and expression levels of the bmpr2 gene by sin3a as a novel therapeutic mechanism in pulmonary arterial hypertension. Circulation.

[33] Wang Y, Huang X, Leng D, Li J, Wang L, Liang Y (2018). DNA methylation signatures of pulmonary arterial smooth muscle cells in chronic thromboembolic pulmonary hypertension. Physiol Genomics.

[34] Joshi SR, Kitagawa A, Jacob C, Hashimoto R, Dhagia V, Ramesh A (2020). Hypoxic activation of glucose-6-phosphate dehydrogenase controls the expression of genes involved in the pathogenesis of pulmonary hypertension through the regulation of DNA methylation. Am J Physiol Lung Cell Mol Physiol.

[35] Yan Y, He YY, Jiang X, Wang Y, Chen JW, Zhao JH (2020). DNA methyltransferase 3b deficiency unveils a new pathological mechanism of pulmonary hypertension. Sci Adv.

[36] Xing XQ, Li B, Xu SL, Zhang CF, Liu J, Deng YS (2019). 5-aza-2’-deoxycytidine, a DNA methylation inhibitor, attenuates hypoxic pulmonary hypertension via demethylation of the pten promoter. Eur J Pharmacol.

[37] Li Q, Wu J, Xu Y, Liu L, Xie J (2019). Role of rasef hypermethylation in cigarette smoke-induced pulmonary arterial smooth muscle remodeling. Respir Res.

[38] Fernandez RA, Wan J, Song S, Smith KA, Gu Y, Tauseef M (2015). Upregulated expression of stim2, trpc6, and orai2 contributes to the transition of pulmonary arterial smooth muscle cells from a contractile to proliferative phenotype. Am J Physiol Cell Physiol.

[39] Wang J, Wu J, Zhu X, Chen J, Zhao J, Xu Y (2021). Absence of the mfg-e8 gene prevents hypoxia-induced pulmonary hypertension in mice. J Cell Physiol.

[40] Li Q, Wang J, Zhu X, Zeng Z, Wu X, Xu Y (2017). Dihydromyricetin prevents monocrotaline-induced pulmonary arterial hypertension in rats. Biomed Pharmacother.

[41] Kosanovic D, Dahal BK, Peters DM, Seimetz M, Wygrecka M, Hoffmann K (2014). Histological characterization of mast cell chymase in patients with pulmonary hypertension and chronic obstructive pulmonary disease. Pulm Circ.

[42] Barberà JA, Peinado VI, Santos S (2003). Pulmonary hypertension in chronic obstructive pulmonary disease. Eur Respir J.

[43] Wang XD, Li F, Ma DB, Deng X, Zhang H, Gao J (2016). Periostin mediates cigarette smoke extract-induced proliferation and migration in pulmonary arterial smooth muscle cells. Biomed Pharmacother.

[44] Wright JL, Zhou S, Preobrazhenska O, Marshall C, Sin DD, Laher I (2011). Statin reverses smoke-induced pulmonary hypertension and prevents emphysema but not airway remodeling. Am J Respir Crit Care Med.

[45] Seimetz M, Parajuli N, Pichl A, Veit F, Kwapiszewska G, Weisel FC (2011). Inducible nos inhibition reverses tobacco-smoke-induced emphysema and pulmonary hypertension in mice. Cell.

[46] Adnot S, Kawut SM (2014). Pulmonary hypertension and emphysema: cure targeting a common cause?. Am J Respir Crit Care Med.

[47] Gredic M, Wu CY, Hadzic S, Pak O, Savai R, Kojonazarov B, et al. Myeloid-cell-specific deletion of inducible nitric oxide synthase protects against smoke-induced pulmonary hypertension in mice. Eur Respir J. 2022;59:2101153. 10.1183/13993003.01153-2021 PMC8989054 34475225

[48] Paulin R, Michelakis E (2012). G-protein-coupled receptors and pulmonary arterial hypertension (pah). Drug Discovery Today: Disease Models.

[49] Cederquist CT, Lentucci C, Martinez-Calejman C, Hayashi V, Orofino J, Guertin D (2017). Systemic insulin sensitivity is regulated by gps2 inhibition of akt ubiquitination and activation in adipose tissue. Mol Metab.

[50] Huang XD, Xiao FJ, Wang SX, Yin RH, Lu CR, Li QF (2016). G protein pathway suppressor 2 (gps2) acts as a tumor suppressor in liposarcoma. Tumour Biol.

[51] Chan S, Smith E, Gao Y, Kwan J, Blum BC, Tilston-Lunel AM (2020). Loss of g-protein pathway suppressor 2 promotes tumor growth through activation of akt signaling. Front Cell Dev Biol.

[52] Si Y, Zhang H, Peng P, Zhu C, Shen J, Xiong Y (2021). G protein pathway suppressor 2 suppresses gastric cancer by destabilizing epidermal growth factor receptor. Cancer Sci.

[53] Spain BH, Bowdish KS, Pacal AR, Staub SF, Koo D, Chang CY (1996). Two human cdnas, including a homolog of arabidopsis fus6 (cop11), suppress g-protein- and mitogen-activated protein kinase-mediated signal transduction in yeast and mammalian cells. Mol Cell Biol.

[54] Lu N, Malemud CJ. Extracellular signal-regulated kinase: a regulator of cell growth, inflammation, chondrocyte and bone cell receptor-mediated gene expression. Int J Mol Sci. 2019;20:3792. 10.3390/ijms20153792 PMC6696446 31382554

[55] Leung HKM, Lo EKK, Chen C, Zhang F, Felicianna, Ismaiah MJ (2023). Zearalenone attenuates colitis associated colorectal tumorigenesis through ras/raf/erk pathway suppression and scfa-producing bacteria promotion. Biomed Pharmacother.

[56] Liu K, Liu XS, Yu MQ, Xu YJ (2013). Change of extracellular signal-regulated kinase expression in pulmonary arteries from smokers with and without chronic obstructive pulmonary disease. Exp Lung Res.

[57] Awad KS, Elinoff JM, Wang S, Gairhe S, Ferreyra GA, Cai R (2016). Raf/erk drives the proliferative and invasive phenotype of bmpr2-silenced pulmonary artery endothelial cells. Am J Physiol Lung Cell Mol Physiol.

[58] Albadawi H, Haurani MJ, Oklu R, Trubiano JP, Laub PJ, Yoo HJ (2013). Differential effect of zoledronic acid on human vascular smooth muscle cells. J Surg Res.

[59] Adikesavan AK, Karmakar S, Pardo P, Wang L, Liu S, Li W (2014). Activation of p53 transcriptional activity by smrt: a histone deacetylase 3-independent function of a transcriptional corepressor. Mol Cell Biol.

[60] Peng YC, Kuo F, Breiding DE, Wang YF, Mansur CP, Androphy EJ (2001). Amf1 (gps2) modulates p53 transactivation. Mol Cell Biol.

[61] Silva CP, Kamens HM (2021). Cigarette smoke-induced alterations in blood: a review of research on DNA methylation and gene expression. Exp Clin Psychopharmacol.

[62] Gao X, Zhang Y, Breitling LP, Brenner H (2016). Tobacco smoking and methylation of genes related to lung cancer development. Oncotarget.

[63] Archer SL, Marsboom G, Kim GH, Zhang HJ, Toth PT, Svensson EC (2010). Epigenetic attenuation of mitochondrial superoxide dismutase 2 in pulmonary arterial hypertension: a basis for excessive cell proliferation and a new therapeutic target. Circulation.

[64] Wang Y, Yan L, Zhang Z, Prado E, Fu L, Xu X (2018). Epigenetic regulation and its therapeutic potential in pulmonary hypertension. Front Pharmacol.

[65] Cheng X, Wang Y, Du L (2019). Epigenetic modulation in the initiation and progression of pulmonary hypertension. Hypertension.

[66] Waxman AB, Elia D, Adir Y, Humbert M, Harari S. Recent advances in the management of pulmonary hypertension with interstitial lung disease. Eur Respir Rev. 2022;31:210220. 10.1183/16000617.0220-2021 PMC9724812 35831007

[67] Piccari L, Allwood B, Antoniou K, Chung JH, Hassoun PM, Nikkho SM (2023). Pathogenesis, clinical features, and phenotypes of pulmonary hypertension associated with interstitial lung disease: a consensus statement from the pulmonary vascular research institute’s innovative drug development initiative - group 3 pulmonary hypertension. Pulm Circ.

[68] Lugg ST, Scott A, Parekh D, Naidu B, Thickett DR (2022). Cigarette smoke exposure and alveolar macrophages: mechanisms for lung disease. Thorax.

[69] Zuo Y, Li B, Gao M, Xiong R, He R, Li N (2024). Novel insights and new therapeutic potentials for macrophages in pulmonary hypertension. Respir Res.

[70] Fan R, Toubal A, Goñi S, Drareni K, Huang Z, Alzaid F (2016). Loss of the co-repressor gps2 sensitizes macrophage activation upon metabolic stress induced by obesity and type 2 diabetes. Nat Med.

[71] Huang Z, Efthymiadou A, Liang N, Fan R, Treuter E (2023). Antagonistic action of gps2 and kdm1a at enhancers governs alternative macrophage activation by interleukin 4. Nucleic Acids Res.

[72] Christou H, Khalil RA (2022). Mechanisms of pulmonary vascular dysfunction in pulmonary hypertension and implications for novel therapies. Am J Physiol Heart Circ Physiol.

[73] Klein J, Diaba-Nuhoho P, Giebe S, Brunssen C, Morawietz H (2023). Regulation of endothelial function by cigarette smoke and next-generation tobacco and nicotine products. Pflugers Arch.

