# Supplementary material for: GPS2 ameliorates cigarette smoking-induced pulmonary vascular remodeling by modulating the ras-Raf-ERK axis
Source: Respir Res. 2024 May 16;25:210. doi: 10.1186/s12931-024-02831-0 (PMC11100185; doi:10.1186/s12931-024-02831-0)
Supplement: Supplementary file 1 — Supplementary Material 1. [file 12931_2024_2831_MOESM1_ESM.docx]

**Supplementary Figures:**


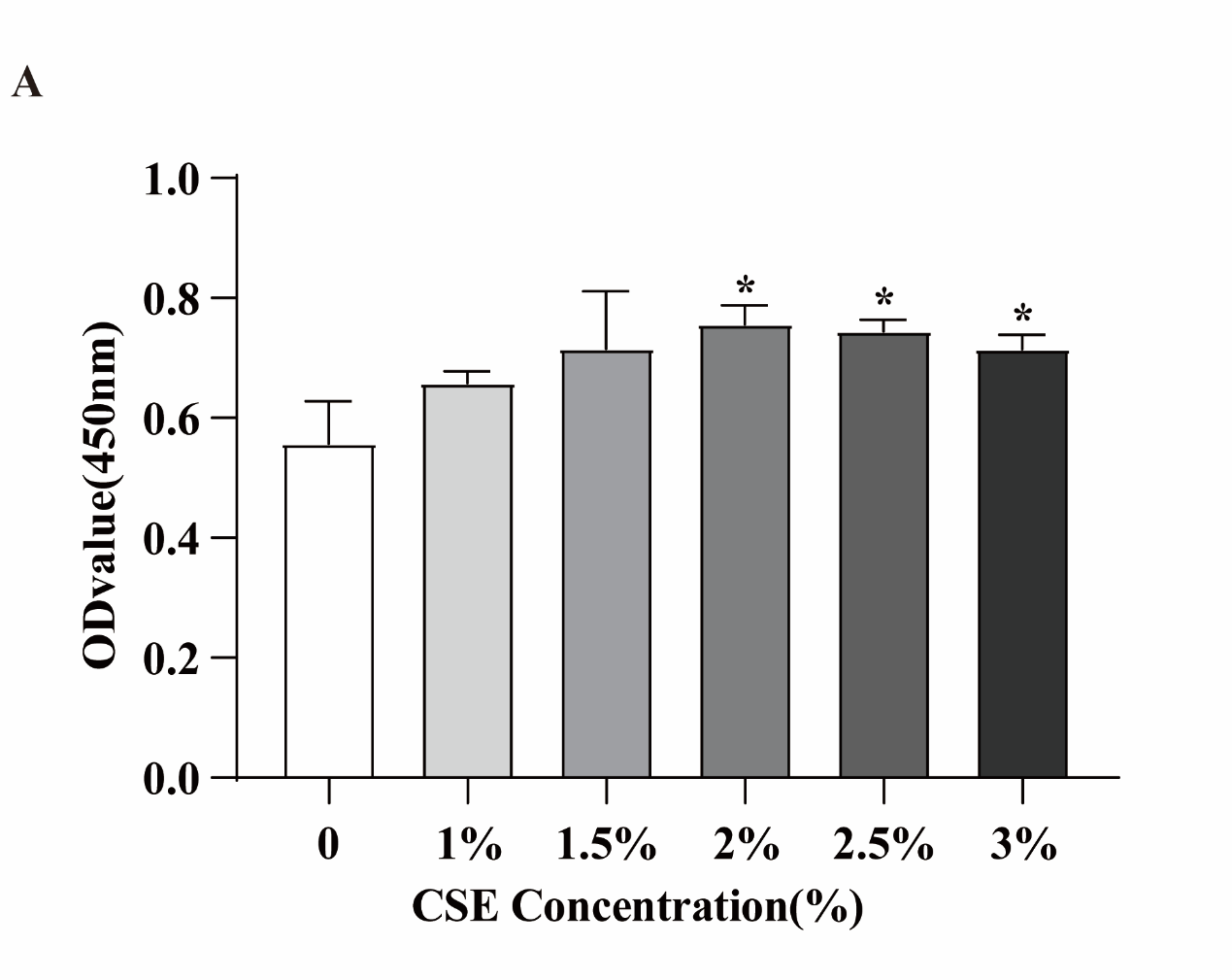


**Figure S1 Cell viability of HPASMCs**

(A) CCK8 assay for the cell viability of HPASMCs after being treated with different concentrations of CSE. (n=3, **P* < 0.05)


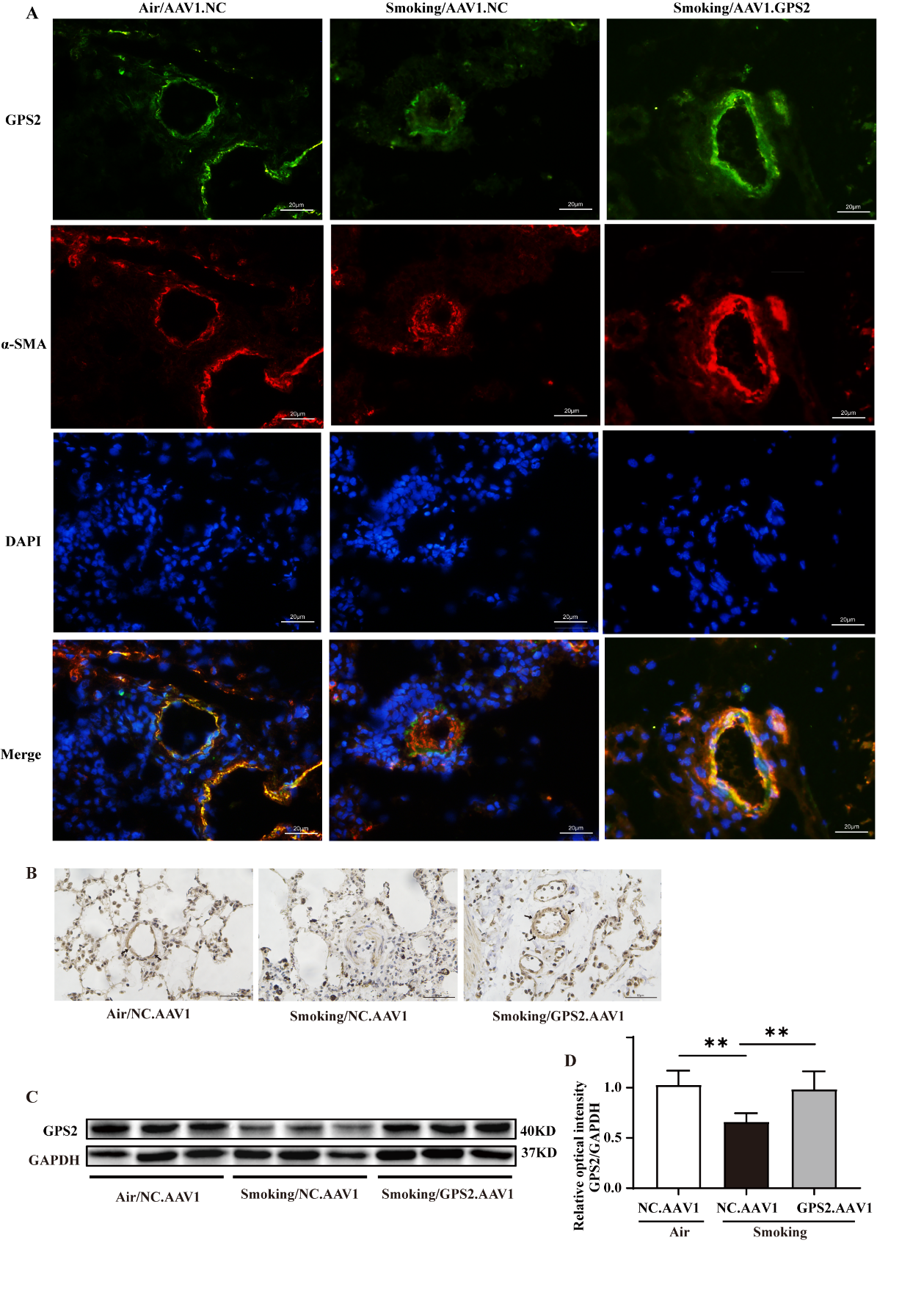


**Figure S2. Effect of adeno-associated virus infection**

**(A)** a-SMA immunofluorescence staining for frozen sections of lung tissue to assess the effect of AAV1 infection in rat PAs (400X, bar=20μm). **(B)** Immunohistochemical assessment of GPS2 expression in rat PAs (400X, arrows indicate GPS2-positive sites, bar=50μm); (C, D) Western blot to detect the expression of GPS2 protein in rat PAs. (n=10, ***P* < 0.01).


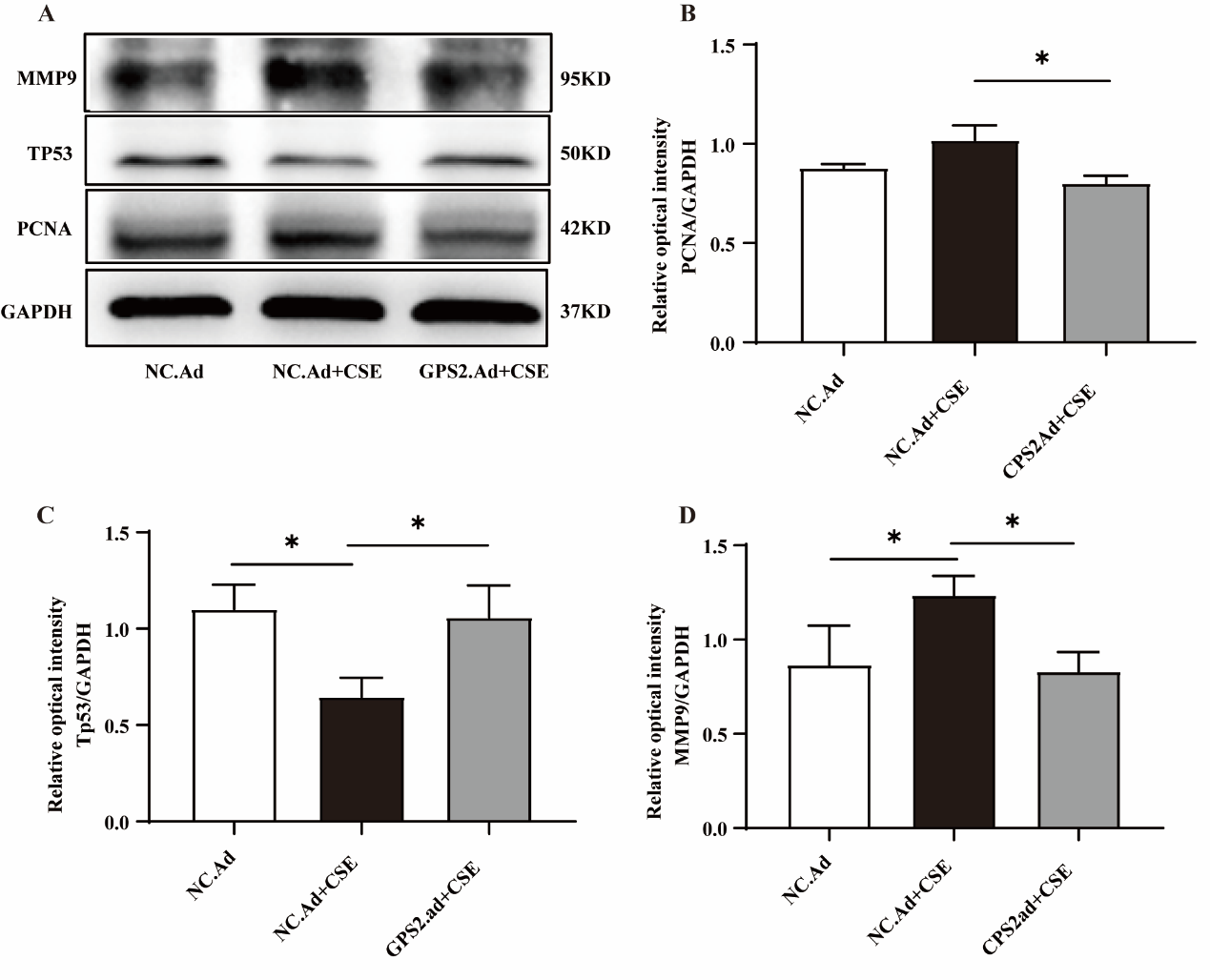


**Figure S3. The expression of function-related proteins in HPASMCs**

**(A-D)** Western blot to detect the expression of function-related proteins in HPASMCs


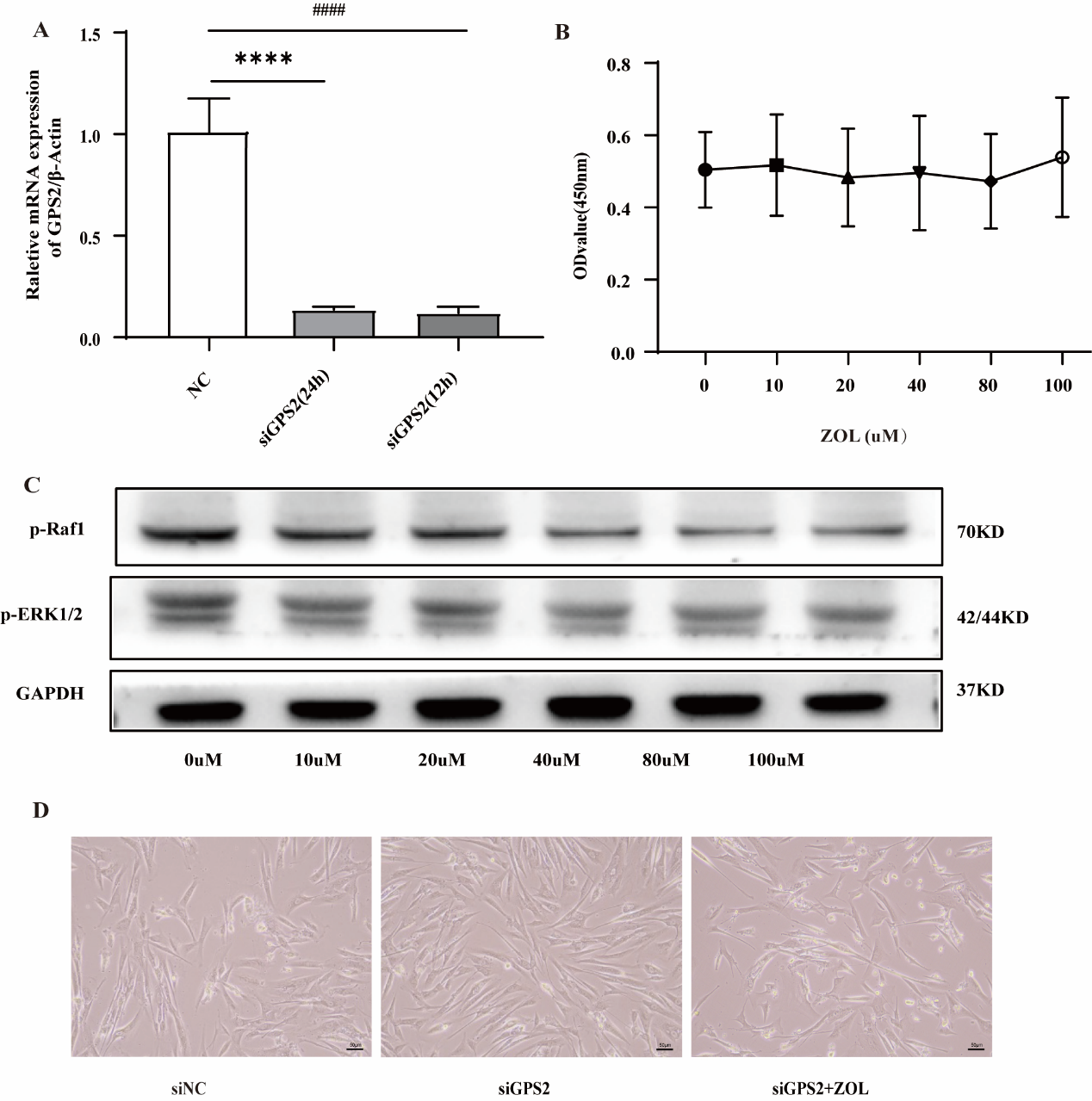


**Figure S4. Effect of ZOL on HPASMCs**

**(A)** Detection of GPS2 mRNA after siGPS2 transfection; (B) CCK8 assay to detect the toxic effects of different concentrations of ZOL on HPASMCs. (C) The activation level of ERK1/2 and Raf1 in HPASMCs after ZOL treatment. (D) the cell confluency of HPASMCs (n=3, ***P < 0.001)


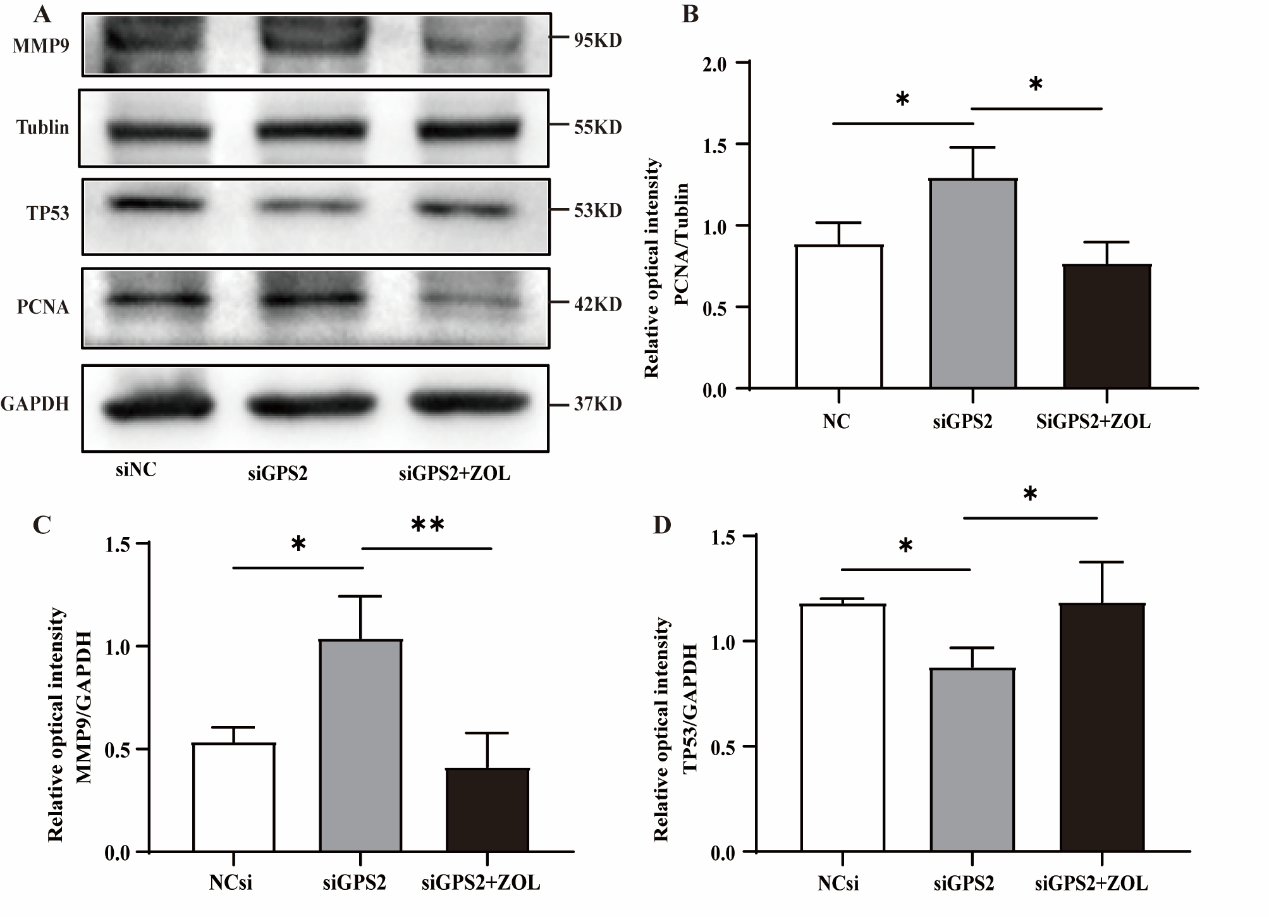


**Figure S5. The expression of function-related proteins in HPASMCs**

(A-D) Western blot to detect the expression of function-related proteins in HPASMCs. (n=3, **P* < 0.05, ***P* < 0.01)
