# Supplementary material for: GPS2 ameliorates cigarette smoking-induced pulmonary vascular remodeling by modulating the ras-Raf-ERK axis
Source: Respir Res. 2024 May 16;25:210. doi: 10.1186/s12931-024-02831-0 (PMC11100185; doi:10.1186/s12931-024-02831-0)
Supplement: Supplementary file 2 — Supplementary Material 2. [file 12931_2024_2831_MOESM2_ESM.docx]

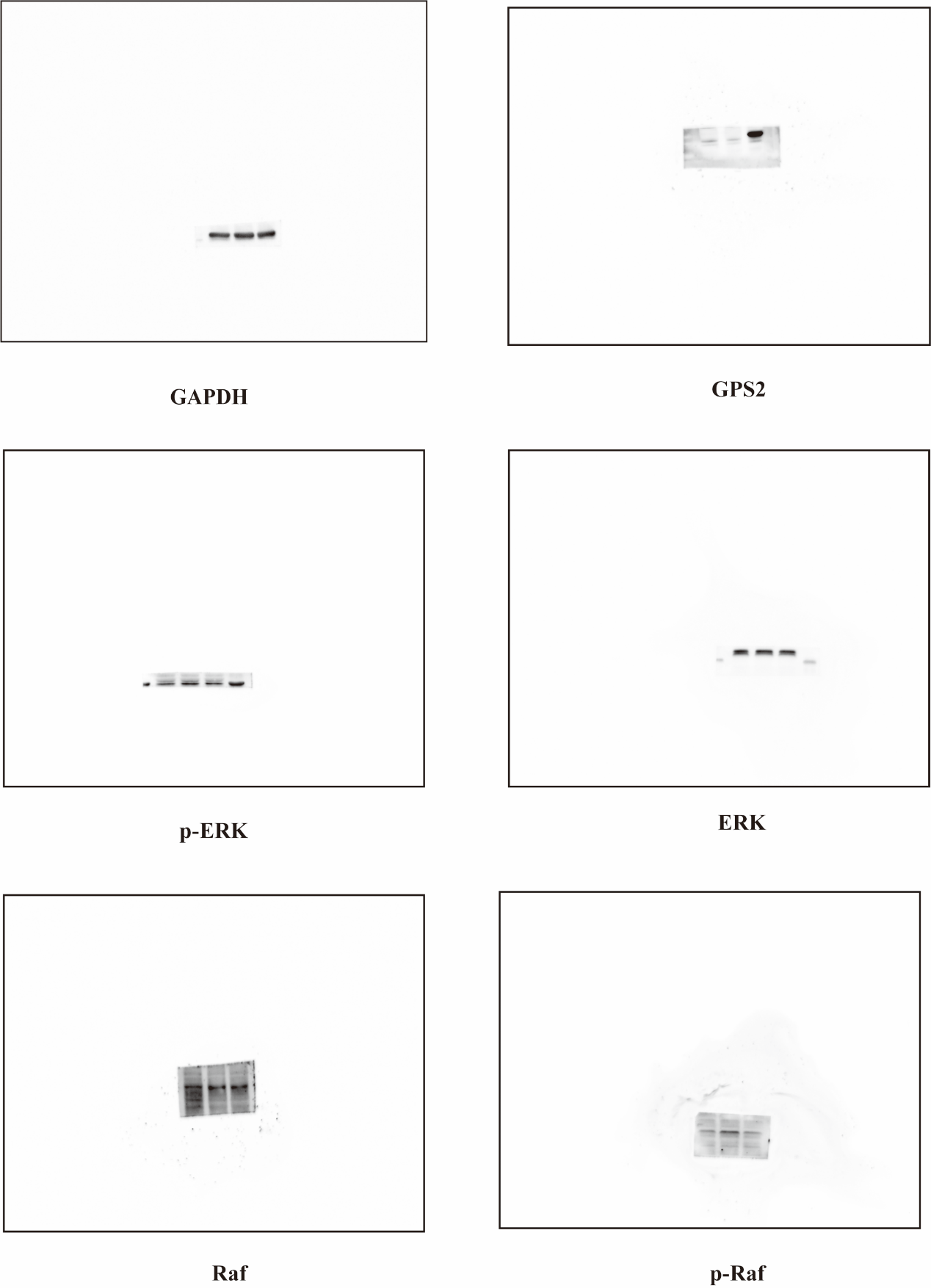


F5 WB original gel photos


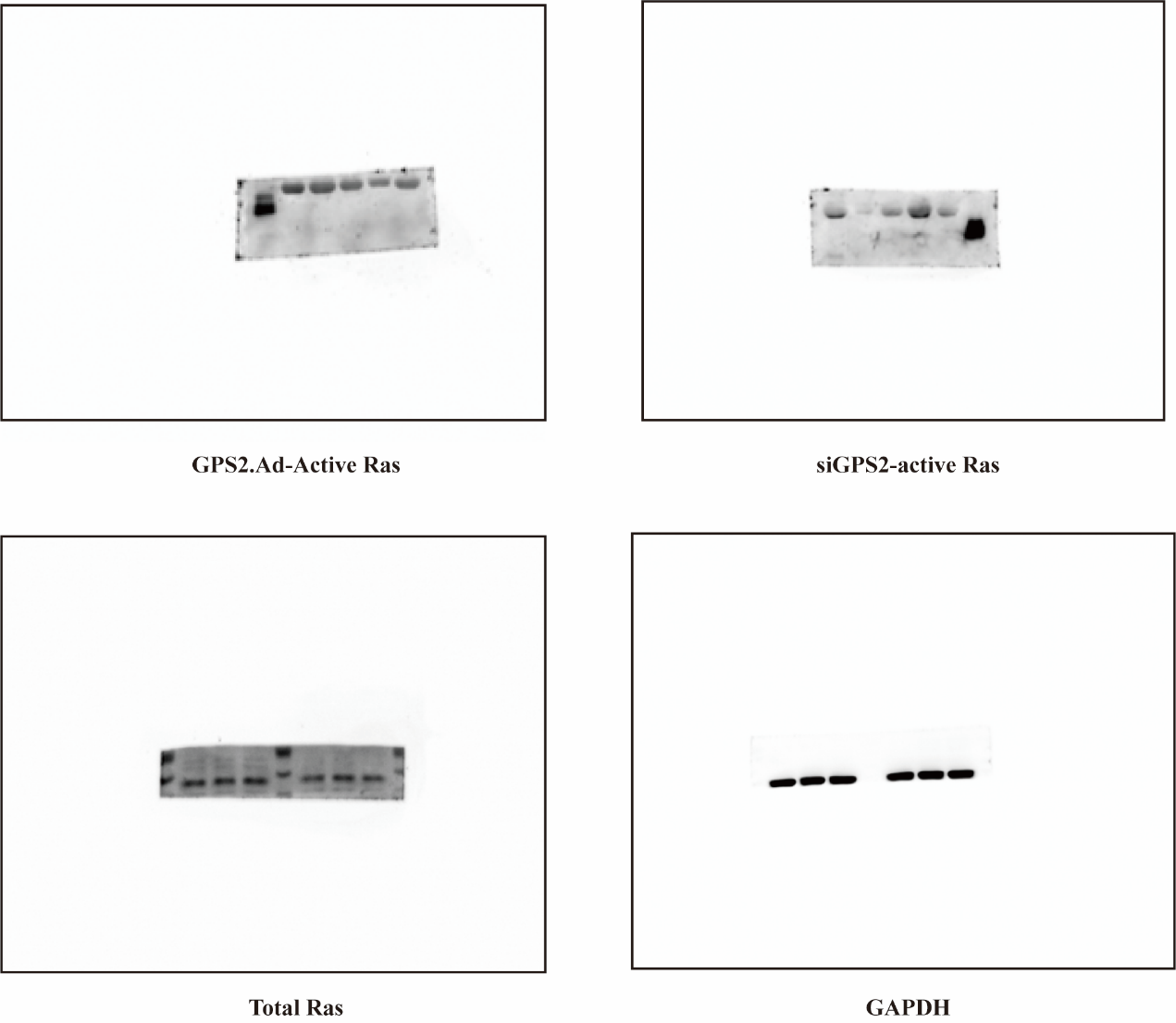


F5 F6 WB original gel photos ,Ras

F6
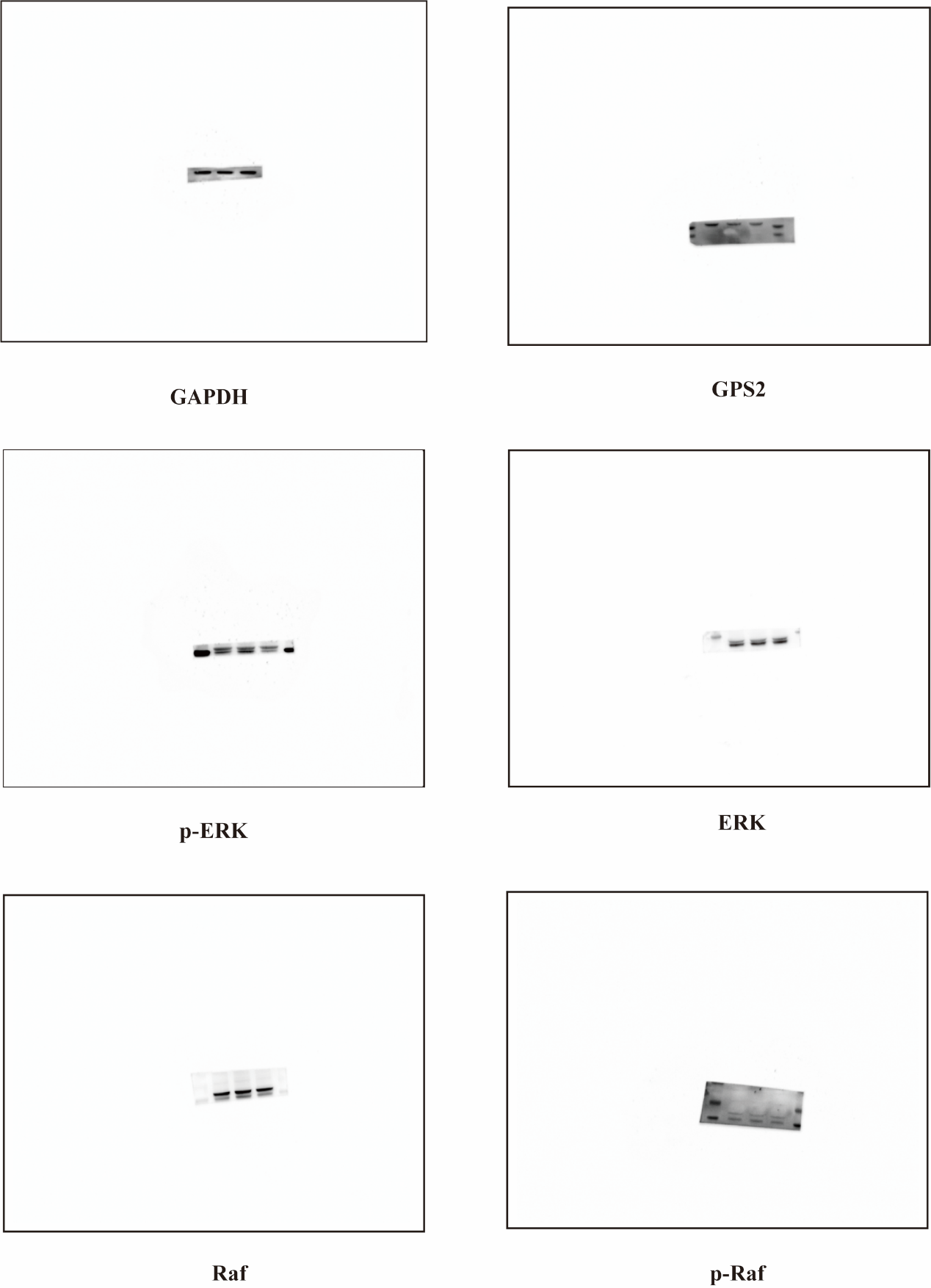


F6 WB original gel photos


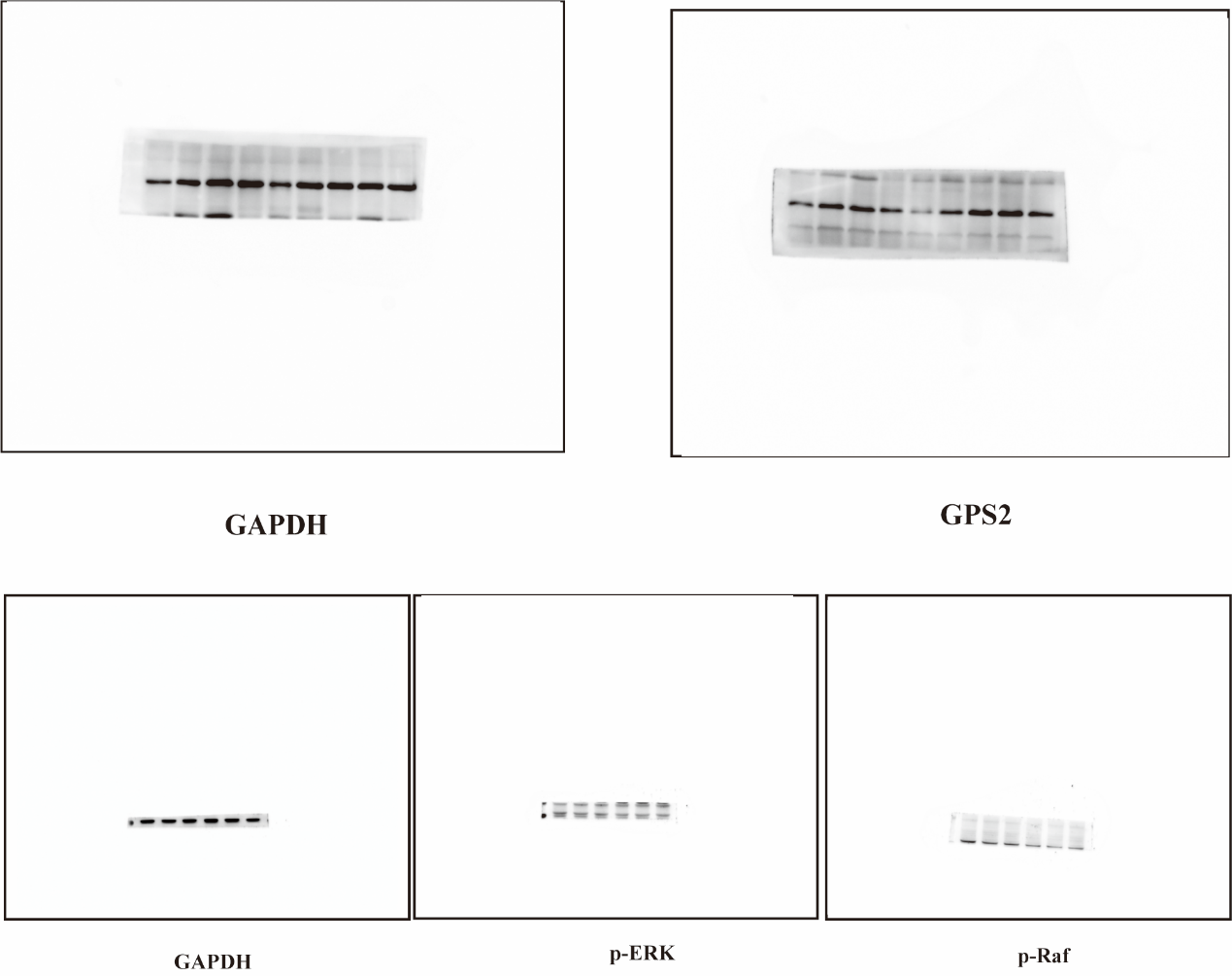


S2, S4 WB original gel photos


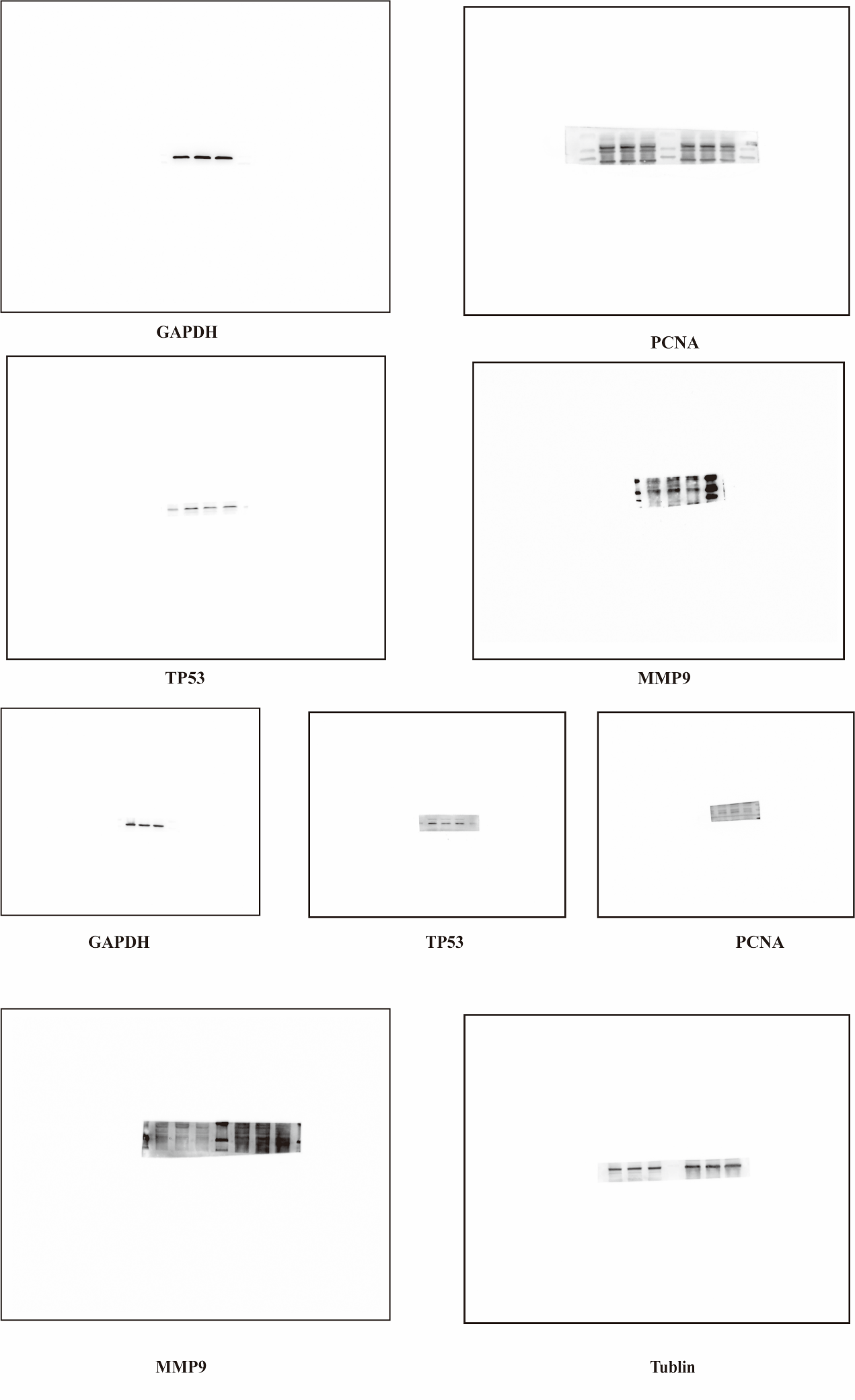


S3, S5 WB original gel photos
